# Sperm-Derived Extracellular Vesicles (Sperm-EVs), Emerging Biomarkers and Functional Modulators in Male Infertility and Assisted Reproduction

**DOI:** 10.3390/genes16121400

**Published:** 2025-11-22

**Authors:** Charalampos Voros, Fotios Chatzinikolaou, Georgios Papadimas, Spyridon Polykalas, Despoina Mavrogianni, Aristotelis-Marios Koulakmanidis, Diamantis Athanasiou, Vasiliki Kanaka, Maria Kanaka, Kyriakos Bananis, Antonia Athanasiou, Aikaterini Athanasiou, Ioannis Papapanagiotou, Dimitrios Vaitsis, Charalampos Tsimpoukelis, Maria Anastasia Daskalaki, Marianna Theodora, Nikolaos Thomakos, Panagiotis Antsaklis, Dimitrios Loutradis, Georgios Daskalakis

**Affiliations:** 1 1st Department of Obstetrics and Gynecology, ‘Alexandra’ General Hospital, National and Kapodistrian University of Athens, 80 Vasilissis Sofias Avenue, 11528 Athens, Greece; depy.mavrogianni@yahoo.com (D.M.); aristoteliskoulak@gmail.com (A.-M.K.); vasilikikanaka@outlook.com (V.K.); maira.kanaka24@gmail.com (M.K.); tsimpoukelischa@gmail.com (C.T.); md181341@students.euc.ac.cy (M.A.D.); martheodr@gmail.com (M.T.); thomakir@hotmail.com (N.T.); panosant@gmail.com (P.A.); gdaskalakis@yahoo.com (G.D.); 2 Laboratory of Forensic Medicine and Toxicology, School of Medicine, Aristotle University of Thessaloniki, 54124 Athens, Greece; fotischatzin@auth.gr (F.C.); loutradi@otenet.gr (D.L.); 3 Athens Medical School, National and Kapodistrian University of Athens, 15772 Athens, Greece; dr.georgepapadimas@gmail.com (G.P.); spyridonpolykalas@hotmail.com (S.P.); gpapamd@hotmail.com (I.P.); vaitsisdim@gmail.com (D.V.); 4 IVF Athens Reproduction Center, 15123 Maroussi, Greece; diamathan16@gmail.com (D.A.); antoathan16@gmail.com (A.A.); diamathan17@gmail.com (A.A.); 5 King’s College Hospitals NHS Foundation Trust, London SE5 9RS, UK; kyriakos.bananis@nhs.net; 6 Fertility Institute-Assisted Reproduction Unit, Paster 15, 11528 Athens, Greece

**Keywords:** sperm extracellular vesicles, epididymosomes, prostasomes, male infertility, capacitation, acrosome reaction, sperm small RNAs, assisted reproduction

## Abstract

Background/Objectives: Approximately 50% of infertility cases are attributable to male factors; yet conventional semen examination can not identify the molecular abnormalities that hinder sperm functionality. Extracellular vesicles (EVs) derived from sperm, such as testicular EVs, prostasomes, and epididymosomes, have become important modulators of oocyte activation, sperm maturation, capacitation, acrosome stability, motility, and early embryonic development. This study aimed to evaluate the potential diagnostic and translational uses of sperm-associated extracellular vesicles (EVs) in male infertility and assisted reproduction, while also consolidating recent insights on their origins, composition, and functional significance. Methods: A focused narrative search of PubMed (2000–2025) was conducted using backward and forward citation tracking. Studies that qualified included human clinical cohorts, functional sperm extracellular vesicle tests, and omics analyses using MISEV-aligned extracellular vesicle isolation and characterisation methodologies. When human mechanistic understanding was constrained, knowledge from animal research was selectively integrated. Results: The cargo signatures specific to the source identified in sperm-derived and seminal EVs encompass proteins, small RNAs, lipids, and enzymatic modules that govern sperm maturation, capacitation, acrosome reaction, redox balance, calcium signalling, zona binding, and DNA integrity. Density-resolved seminal extracellular vesicle subfractions (EV-H/EV-M/EV-L) have unique functional and proteomic characteristics linked to progesterone-induced hyperactivation, oxidative stress, and motility. Asthenozoospermia and oligoasthenoteratozoospermia are associated with changes in extracellular vesicle composition, reduced embryonic developmental potential, compromised oocyte activation (related to PLCζ), and increased sperm DNA fragmentation. Numerous EV-related miRNA and protein signatures may predict TESE results, identify functional sperm anomalies not recognised by conventional semen analysis, and differentiate between obstructive and non-obstructive azoospermia. Conclusions: The available findings indicate that sperm-derived extracellular vesicles are significant functional regulators of sperm physiology and may serve as valuable non-invasive indicators for male infertility. The standardisation of EV isolation, characterisation, and clinical validation is essential prior to widespread use; nonetheless, their integration into liquid biopsy methods and assisted reproductive technology processes represents a significant improvement.

## 1. Introduction

This overview guides the reader from the beginnings of vesicles to fraction-resolved processes and clinical consequences, using concise signposts and species tags to maintain clarity throughout the narrative.

### 1.1. Male Infertility: The Unaddressed Requirement Beyond Semen Analysis

Routine semen analysis of concentration, motility, and morphology serves as the initial clinical assessment for the male partner. However, its descriptive characteristics do not encompass the molecular deficiencies that ultimately affect fertilisation capability and embryonic developmental potential [1]. A considerable number of men with infertility have “normal” fundamental parameters, while those with aberrant semen analysis findings successfully attain fertilisation and pregnancy highlighting a discrepancy between observable phenomena and underlying causes. To address this disparity, it is imperative to integrate the molecular biology and pathology of the male gamete, with particular emphasis on sperm-derived signalling systems and the Extracellular Vesicles (EV) networks that govern sperm functionality along the testis–epididymis–accessory gland axis and within the female reproductive tract [2].

Oocyte activation exemplifies a crucial biological factor that remains imperceptible. After the union of sperm and oocyte membranes, a certain pattern of cytosolic Ca^2+^ oscillations in the oocyte is essential and adequate to revive meiosis and initiate embryogenesis [3]. In humans, these Ca^2+^ oscillations are triggered by sperm-derived Phospholipase C zeta (PLCζ), the most recognised sperm-borne oocyte-activating factor (SOAF). Mutations in PLCZ1 that result in its absence, misplacement, or functional loss are significantly associated with fertilisation failure or suboptimal fertilisation following ICSI, despite normal routine semen parameters [4]. Both quantitative and qualitative aberrations of PLCζ have been recognised in infertile men; moreover, pathogenic mutations of PLCZ1 have been linked to oocyte activation deficit (OAD), including in men with normozoospermia. These findings clarify the reasoning for the potential of artificial oocyte activation (AOA) to “rescue” cycles previously marked by total fertilisation failure, alongside the examination of PLCζ assays and selection/enrichment strategies for PLCζ-positive sperm as precise tools, none of this can be inferred from a standard semen analysis report [5].

The paternal contribution to early embryogenesis encompasses oocyte activation, chromatin packaging, retained histones, and sperm-derived RNAs that influence zygotic transcriptional programmes. Spermatogenesis involves significant protamination that condenses the genome, while some areas retain histones with epigenetic modifications thought to affect early developmental trajectories [6]. Post-fertilisation, these programmed characteristics are dynamically reconfigured and may be influenced by disease. For instance, anomalous protamine ratios, irregular histone retention, or dysregulated non-coding RNAs can impede embryo development without altering sperm quantity or motility. Conventional semen analysis fails to identify these lesions [7].

Significantly, many molecular attributes are not intrinsic to testicular sperm, they are altered post-testicularly by extracellular vesicles. EVs are lipid-bilayer nanoparticles secreted in the male reproductive tract (testicular vesicles, epididymosomes (mouse), and prostate-derived prostasomes (humans)) and in the female reproductive tract (oviductal and endometrial EVs in human or cats) [2]. They transport proteins, lipids, and regulatory RNAs, and they either fuse with or attach to sperm to modify their surface and contents. This regulates maturation, capacitation preparedness, acrosome reactivity, and immune evasion. Epididymal extracellular vesicles modify the small RNA composition of sperm as they traverse the epididymis. This process resembles that in rats and significantly influences the flexibility of the human sperm epigenome [8].

Seminal extracellular vesicles display molecular and biophysical variability that correlates with their functionality. High-resolution iodixanol gradients have been employed to categorise seminal extracellular vesicles into three classifications: high-density (EV-H), medium-density (EV-M), and low-density (EV-L) in humans. Every group possesses an own array of markers and proteins [9]. NV could change how cholesterol is handled in membranes, which might have an indirect effect on motility. However, it has not yet been shown that NV consistently improves motility directly. EV-H can aid with capacitation-related motility readouts. GSTM2 makes EV-M a powerful antioxidant, which decreases the quantity of ROS in sperm. An imbalance of ROS is a prevalent factor in unexplained male infertility and DNA damage; thus, EV-mediated redox buffering serves as a crucial molecular safeguard that, while not observable in semen counting chambers, is directly correlated with fertilisation capability [10].

Prostasomes in human exosome like extracellular vesicles originating from the prostate further illustrate the impact of accessory gland biology on sperm functionality. Prostasomes encompass Ca^2+^-signalling constituents (including cADPR machinery and proteins associated with Ca^2+^ regulation) that sperm can acquire to achieve progesterone-induced hyperactivation, they also house enzymes and modulators that regulate the timing of capacitation, acrosome responsiveness, and motility. Disruption of these EV-sperm interactions or qualitative changes in prostasome cargo may result in “unexplained” fertilisation problems, despite normal semen parameters [11].

EVs are not merely transport vehicles; they are modulators that adapt according to the conditions within the female reproductive system. Subsequent to insemination, sperm interact with oviductal and uterine EVs that selectively adhere to the acrosome/midpiece. These EVs can improve motility, stabilise acrosome integrity, and potentially transport vital membrane proteins, such as PMCA4 (mouse), a primary sperm Ca^2+^ efflux pump whose presence facilitates appropriate motility patterns. EVs facilitate immunological tolerance to foreign substances by attenuating the activity of NK cells and modulating leukocyte behaviour [12].

From a pathological standpoint, the EV domain has initiated the examination of disease-related markers. The proteome and RNA profiles of seminal EVs differ between fertile individuals and men with asthenozoospermia or other semen anomalies. Particular extracellular vesicle fractions can variably affect capacitation, the acrosome response, and oxidative stress in vitro [13].

Two primary clinical implications exist. Initially, molecular diagnoses such as OAD (PLCζ-related), redox dysregulation, or impaired Ca^2+^ signalling may coexist with “normal” semen parameters. Identifying these lesions necessitates molecular studies (such as PLCζ quantification/localisation and small-RNA/cargo profiling) and functional assessments (including sperm-induced Ca^2+^ oscillations and EV sperm fusion experiments) [14]. Descriptive microscopy is insufficient on its own. Secondly, EVs may serve as non-invasive biomarkers and functional modulators. Their prevalence, subtype equilibrium, and cargo composition in semen provide a “liquid biopsy” of the male reproductive system. Researchers are investigating the utilisation of exogenous or modified extracellular vesicles to enhance sperm efficacy in assisted reproductive technology workflows, for as by including antioxidants or transporting signalling proteins. Recent systematic studies consolidate these concepts, asserting that extracellular vesicle biology should be incorporated into male-factor evaluations and assisted reproductive technology decision-making [15].

Environmental factors exacerbate the molecular structure of male-factor infertility. Recent findings reveal that semen is particularly susceptible to environmental pollutants, such as endocrine disruptors and airborne particulates, which may directly affect the biochemical characteristics of sperm nuclear basic proteins. These xenobiotics cause oxidative damage to nucleoproteins, irregular transitions of protamines and histones, and altered chromatin compaction, all of which are undetectable by standard semen examination. Moreover, seminal microRNA profiles are significantly altered in males living in polluted areas, with pollutant-responsive miRNAs influencing pathways related to mitochondrial stress, apoptosis, and DNA repair. EVs derived from both sperm and seminal plasma may convey environmentally modified short RNAs. These alterations engage with the EV-mediated signalling axis to transmit pollutant-induced molecular markers to the oocyte and early embryo. The results indicate that pollution exposure may inexplicably lead to infertility and acts as a clinically important modulator of sperm extracellular vesicle cargo [16,17].

### 1.2. What Are Extracellular Vesicles?

EVs are lipid-bilayer particles measuring from nanometres to micrometres, secreted by nearly all cell types. They contain proteins, lipids, and nucleic acids that are specifically encapsulated. Within the testis–epididymis–accessory gland axis, they are synthesised in the male reproductive system and accumulate in elevated concentrations in semen. They engage with spermatozoa and cells of the female reproductive tract. It is infrequently feasible to ascertain whether vesicles derive from endosomes (“exosomes”) or the plasma membrane (“microvesicles”) in intricate biofluids. Thus, contemporary nomenclature consistent with MISEV classifies vesicles according to size and biophysical characteristics (e.g., small vs. large EVs) and origin terminology we employ throughout (e.g., epididymosomes, prostasomes, seminal plasma EVs, “spEVs”) [18].

The diversity of extracellular vesicles in semen is considerable and biologically important. High-resolution iodixanol gradients segregate human seminal extracellular vesicles into a minimum of three density-defined subtypes EV-H, EV-M, and EV-L. Each subtype possesses a distinct morphology, marker profile, and proteomic composition. A non-vesicular nanoparticle (NV) layer co-isolates unless explicitly resolved [19]. Extracellular vesicles, known as NV, lack a surrounding lipid bilayer. Instances of NV encompass protein/protein–RNA complexes and lipoproteins such as ApoA1-positive HDL and several other lipoprotein particles. Following precipitation or differential ultracentrifugation, NV often co-isolates with small-EV pellets; size-exclusion chromatography and/or iodixanol density gradients enable NV depletion. In contrast to EVs (CD9/CD63/TSG101/ALIX-positive), NVs are often negative for EV markers and frequently include higher levels of ApoA1/ApoB and soluble proteins. The EV-H and NV fractions facilitate sperm motility and capacitation in vitro. The EV-M fraction exhibits greater antioxidant activity, similar to GSTM2, which inhibits intrinsic sperm ROS. The EV-L fraction exhibits a “small-tubule” morphology observable by cryo-electron microscopy and may aggregate lipid-rich entities. These fraction-specific phenotypes demonstrate that “bulk exosome” readouts may hide conflicting biological data and that NV contaminants might hamper both mechanistic and biomarker investigations if not eradicated [20].

Two populations originating from a specific source are responsible for sperm-related signals. Epididymosomes, originating from the epididymal epithelium and discharged into the luminal fluid, attach to and temporarily fuse with sperm traversing the body, thereby altering the sperm’s surface and cargo [21]. Redox enzymes and conserved proteins, such as the CRISP family, ADAM metalloproteases, MIF, and chaperonins, are localised to certain regions of sperm, aligning with the sperm’s motility, preparation for fertilisation, binding to the zona, and acrosomal competence [22]. Epididymosomes transport several short RNAs (miRNAs and tRFs) that are incorporated into sperm and contribute to embryonic development in model organisms. This EV-guided maturation landscape is recognised for altering the lipids in the sperm membrane from caput to cauda, incorporating sphingomyelin and cholesterol. Pathologically, deviations in the composition or delivery of epididymal extracellular vesicles (e.g., diminished antioxidant transfer, altered small-RNA profiles) provide a molecular basis for asthenozoospermia, DNA damage, and premature developmental arrest that cannot be inferred from conventional semen measures [23].

Prostasomes, the predominant kind of prostate-derived extracellular vesicles, illustrate the influence of accessory gland biology on sperm functionality and immune defence. Their membranes and payload are designed for signalling post-ejaculation. They provide “Ca^2+^-signalling tools” (such as cADPR machinery and Ca^2+^-handling proteins) essential for progesterone-induced motility and hyperactivation [24]. They can also modulate capacitation trajectories (such as tyrosine-phosphorylation dynamics) and direct delivery to the neckpiece, aligning with the regulation of flagellar behaviour in certain regions. Prostasomes exhibit immunological activity, harbouring complement regulators (CD46, CD59) and NK-cell-modulatory ligands (e.g., CD48) that safeguard sperm from complement-mediated damage and diminish leukocyte activity in the female reproductive tract functions directly linked to unexplained fertilisation failure and post-coital immune infertility [25]. Disruptions in these pathways (e.g., reduced Ca^2+^ transport, zinc-dependent motility inhibition, impaired immunological crosstalk) indicate molecular diseases that are undetectable by the trio of count, motility, and morphology.

Their tetraspanin-enriched membranes (e.g., CD9/CD63) and adhesion mechanisms enable sperm to selectively attach to the head, neck, or midpiece, subsequently leading to endocytosis or membrane fusion. In the female reproductive system, oviductal and uterine EVs can transport PMCA4 and SPAM1 to sperm in mouse, hence maintaining calcium homeostasis, enhancing cumulus penetration and optimising capacitation. The receptor-mediated uptake and cargo transfer provide a biological basis for the translation of external inputs into alterations in sperm signalling pathways (cAMP/PKA, Ca^2+^ flow, tyrosine phosphorylation) that promote hyperactivation and acrosome preparedness. The dysfunction of these EV-mediated exchanges stemming from altered ligand-receptor interactions, aberrant EV synthesis, or impaired cargo offers a pathophysiological explanation for otherwise “idiopathic” fertilisation problems [25].

Heterogeneity impacts both diagnosis and translation. Proteomic and RNA analyses have consistently shown that the cargo of seminal EVs differs between fertile men and those with asthenozoospermia, with specific candidates (e.g., TRPV6 levels in spEVs) correlating with calcium regulation and motility characteristics; region-specific EVs along the tract display unique marker constellations and biophysical signatures, underscoring the importance of distinguishing and reporting EV subtypes rather than combining them [26]. Modern single-EV analytics, including imaging flow cytometry, are employed for epididymal and seminal vesicle EVs to clarify population structure, reduce co-isolation artefacts (lipoproteins, protein aggregates), and align fraction identity with function crucial prerequisites for dependable biomarker development and clinical assay reproducibility.

EV-mediated signalling transpires primarily at the interface between sperm and the female reproductive system, including the cervix, uterus, oviduct, and cumulus-oocyte complex. EVs derived from seminal and tract sources convey immunoregulatory, redox, and calcium-regulating elements (e.g., complement regulators, GSTM2, PMCA4) that protect sperm functionality. However, inflammation or infection may compromise this interface, obstructing fertilisation. In semen, EV fractions that fail to adequately buffer ROS due to the loss of GSTM2-mediated protection exacerbate oxidative DNA damage and impede embryo development; EVs that insufficiently supply calcium-handling mechanisms compromise progesterone responsiveness and trigger hyperactivation; and immune-modulatory deficiencies render sperm vulnerable to complement and NK cell assault. Conversely, excessive or inopportune EV signals may induce premature capacitation or acrosome exocytosis, thus disrupting the timing of fertilisation. Identifying EVs as dynamic, source-specific, fraction-specific regulators rather than a unique “exosome” pool advances molecular biology and pathology in male-factor infertility and enables practical approaches for liquid-biopsy diagnostics and targeted adjuvants in ART.

### 1.3. Objectives and Aims

A strictly cellular viewpoint on sperm functionality does not clarify various molecular phenotypes identified in male infertility clinics, including disordered capacitation despite normal sperm counts, fertilisation failure after ICSI with morphologically “normal” sperm, or inconsistent DNA integrity in men with otherwise stable semen profiles. EVs emitted from the male reproductive tract (testis, epididymis, accessory glands) and by sperm introduce a novel, dynamic signalling layer that likely links tract physiology to sperm molecular remodelling and, ultimately, reproductive outcomes [2]. Seminal EVs display heterogeneity in size, content, and biogenesis, resulting in the creation of subfractions defined by unique proteomes and functional properties. Density-resolved seminal extracellular vesicle subgroups (EV-H, EV-M, EV-L) exhibit differences in protein and RNA content, which correlate with sperm motility and oxidative stress indicators in clinical specimens. This indicates a correlation between EV composition and sperm functionality.

Based on research, EVs convey regulatory short RNAs and proteins to sperm before and after epididymal transit, altering the sperm epigenome, surface proteome, and signalling preparedness. Seminal exosomes possess a distinctive collection of short non-coding RNAs expected to play roles in gene regulation, providing a promising source for biomarkers and possible modulators of sperm function [27].

In both human and animal research, specific EV-associated miRNAs are connected with motility and apoptotic pathways. For example, miR-222, sourced from semen extracellular vesicles, inhibits BCL2L11-mediated apoptosis in sperm, thereby demonstrating a causal link between extracellular vesicle cargo and sperm physiology. Epididymal extracellular vesicles (epididymosomes) modify the sperm surface throughout maturation [28]. Proteomic analysis throughout the epididymis indicates the incorporation of proteins linked to motility, zona recognition, and immunological interactions, particularly the pathways that are lacking in certain idiopathic instances.

The molecular exchanges are not simply developmental relics, pure CD63-positive epididymosomes derived from human semen can substantially improve sperm functioning in vitro, highlighting their translational potential. EV signalling likely extends beyond the male reproductive tract and into the vicinity of conception. Seminal EVs modulate the activity of antigen-presenting cells and inhibit antiviral T-cell responses, suggesting an immune-tolerising role relevant to implantation [29]. They can initiate immune-related gene programmes in the endometrium and facilitate decidualisation, suggesting a coordinated male-to-female tract connection that may affect implantation success and the risk of miscarriage. This dialogue is vulnerable from a pathological standpoint: infection, inflammation, or endocrine disruptors may modify EV cargo and potentially cause malfunction in sperm or the endometrium, despite semen parameters appearing “normal” [30].

Clinically, extracellular vesicles present two immediate applications. Initially, as liquid-biopsy biomarkers, seminal extracellular vesicle miRNA and lncRNA signatures classify the origin of azoospermia and predict testicular sperm retrieval. Standard semen analysis identifies asthenozoospermia; however, asthenozoospermic and normozoospermic males display unique molecular signatures, as evidenced by seminal extracellular vesicle proteomics (e.g., TRPV6↓ and other candidate proteins in asthenozoospermic extracellular vesicles; CRISP1↑ and glycodelin↓ in normozoospermic exosomes), which provide mechanistic insights and prospective biomarkers [31]. Secondly, as functional modulators: the incorporation of specific EV preparations enhances sperm quality post-thawing in preclinical models and may mitigate cryodamage, a prevalent issue in male fertility preservation and ART protocols [32].

Although the significant potential of EVs, they are not routinely incorporated into andrology or ART protocols. The technical diversity in EV isolation and characterisation, the incomplete identification of EV sources (testicular, epididymal, prostatic, or sperm-derived), and the restricted direct clinical validation against gold-standard outcomes (fertilisation rate, blastulation, euploidy, live birth) have hindered clinical implementation [33]. Recent advancements in fractionation and nanoscale phenotyping have enabled the assignment of functions to specific EV subsets and the correlation of their cargo with distinct sperm pathways (motility, capacitation, acrosome readiness, apoptosis), thereby facilitating assays and interventions suitable for clinical applications [34].

This review has three objectives. Initially, to incorporate current knowledge concerning the cellular origins, production, and composition of sperm-derived and seminal EVs, emphasising their function in the reconfiguration of the sperm proteome and regulatory RNA landscape throughout maturation and ejaculation. Secondly, to elucidate EV-mediated mechanisms including protein transfer, membrane fusion, receptor/tetraspanin interaction, and small-RNA delivery and their correlation with distinct sperm functions (maturation, capacitation, acrosome reaction, motility, and DNA stability) as well as the immune and endometrial responses in females that affect implantation. Third, to rigorously assess translational applications: (i) diagnostic signatures for male-factor subtypes (e.g., OAT, NOA, recurrent pregnancy loss), (ii) prognostic instruments for ART decision-making (ICSI vs. IVF, necessity for AOA, likelihood of testicular sperm), and (iii) interventional strategies utilising native or engineered EVs to enhance sperm quality (e.g., cryoprotection, apoptosis inhibition, membrane remodelling), while specifying the analytical and clinical validation processes required for implementation.

We aim to progress from basic descriptive semen analysis to mechanism-based diagnostics and therapies by integrating extracellular vesicle biology with clinical outcomes. The working hypothesis asserts that specific extracellular vesicle subpopulations and their corresponding cargo function as measurable indicators of sperm vitality and as adjustable mechanisms to rehabilitate functioning in male-factor infertility and to customize ART.

Recent study has shown a correlation between alterations in seminal and sperm-derived extracellular vesicles and many significant medical problems. OAT, non-obstructive azoospermia, and varicocele-associated hypoxia have all been shown to exhibit modifications in EV cargo, density fractions, or functional activity. These alterations are often associated with compromised motility, capacitation, DNA integrity, or fertilisation competence. Viral and inflammatory conditions, including as prostatitis, leukocytospermia, and high-risk HPV infection, might alter seminal extracellular vesicle profiles. These ailments impair sperm functionality and the development of early embryos. Systemic or environmental factors, including pollution, obesity, smoking, febrile illness, and endocrine dysregulation, further affect EV-mediated signalling pathways and small RNA transfer. The disease-specific extracellular vesicle signatures indicate that sperm-derived extracellular vesicles may serve as non-invasive diagnostics for male reproductive disorders and as underlying mechanisms of infertility.

## 2. Methods

We give an overview of MISEV-consistent EV handling and PRISMA-aligned selection here. The main text keeps the reasoning and decision rules, and supplementary methods has all the details about the procedures.

### 2.1. Sources of Information and Search Methodologies

We conducted a focused narrative search utilising PubMed as the primary resource. We conducted backward and forward citation chaining from existing seed articles and intermittently supplemented our research based on warnings during the drafting process. The emphasis was on human studies for clinical endpoints, with selective reference to animal studies when mechanistic understanding in humans is limited (e.g., epididymal EV cargo transfer, small-RNA remodelling, recipient-site tropism), concentrating on EVs in semen and sperm and their relevance to male infertility and assisted reproductive technology (IVF/ICSI). The selected date range of 1 January 2000 to 31 August 2025 encompasses the contemporary molecular era of EV biology and assisted reproduction. Nonetheless, sentinel data on prostasomes and seminal physiology before to 2000 may be incorporated if required for mechanistic purposes. Only English records were examined.

The queries employed both MeSH and free-text terminology, subsequently refined repeatedly to enhance their sensitivity and specificity. The primary Boolean string was (“extracellular vesicles” OR exosome* OR “small extracellular vesicle*” OR microvesicle* OR epididymosome* OR prostasome* OR “seminal plasma extracellular vesicle*”). (sperm OR spermatozoa OR semen OR “seminal plasma”) AND (infertility OR “male infertility” OR “assisted reproductive technology*” OR IVF OR ICSI OR capacitation OR acrosome OR hyperactivation OR CatSper OR PMCA4 OR PLCZ1 OR “oocyte activation” OR “DNA fragmentation” OR oxidative OR ROS OR azoospermia OR “testicular sperm extraction” OR TESE OR NOA OR asthenozoospermia OR OAT). We used terminology such as biomarker, “liquid biopsy,” proteomic, miRNA, “tRNA fragment*,” metabolomic, as well as methodological terminologies like dUC, DG, SEC, IFC, NTA, TEM, cryo-EM, DLS, “density fractionation,” “iodixanol gradient,” and MISEV to enhance therapeutically relevant outcomes and improve molecular research.

We examined the abstracts and titles for EV isolation and characterisation, as well as explicit connections to sperm and seminal environment. We retained complete texts that documented clinical endpoints (fertilisation rate, blastulation, pregnancy/live birth, TESE success) or functional assessments pertaining to sperm (motility, capacitation markers, acrosome status, zona binding, ROS/DFI, fertilisation competence) or isolated extracellular vesicles employing established methodologies (dUC, DG, SEC, size-based or immuno-capture) and included at least two orthogonal characterisations (e.g., TEM/cryo-EM alongside particle sizing and immunomarkers). We prioritised studies that concentrated on NV co-isolation and, when feasible, included subfraction analyses (e.g., EV-H/EV-M/EV-L in humans) rather than bulk pellets, owing to the substantial presence of non-vesicular nanoparticles in semen. Leukocytospermia and the abstinence interval were identified as potential confounders during screening and employed to assess research comparability.

We employed a systematic approach to snowballing: for each paper included, we examined reference lists and “cited by” links to remain abreast of novel methodologies (IFC, single-EV phenotyping) and to identify pertinent primary research (e.g., cargo transfer specific to the epididymal region, prostasome–sperm Ca^2+^ signalling, spEV subfraction bioactivity). The biofluid or cellular source, isolation and characterisation workflow, extracellular vesicle subtype or fraction (including EV-H/EV-M/EV-L where applicable), cargo classification (proteins, miRNAs, tRFs, metabolites, and lipids), mapped recipient region on sperm (head/neck/flagellum when available), functional assay(s) and endpoint(s), clinical cohort descriptors, key effect sizes or directional findings, and articulated limitations were all derived from studies that fulfilled the eligibility criteria.

Each search session was documented with the date and time, precise Boolean strings, filters, and tallies of entries that were discovered, evaluated, and retained for future reference. We maintained a PRISMA-style flow, documenting the rationale for study exclusions and maintaining a dynamic inclusion list associated with the part where each study is ultimately referenced, despite the synthesis being narrative rather than meta-analytical.

### 2.2. Inclusion and Exclusion Criteria

We incorporated research that investigated EVs within a sperm/seminal framework and correlated them with molecular or clinical markers relevant to male infertility or ART. Eligible designs encompassed human observational cohorts, case–control studies, interventional or functional assays (EV add-back, fraction bioassays), and omics investigations (proteomic, small-RNA, metabolomic, lipidomic), contingent upon the isolation of EVs using established methodologies (dUC, DG, SEC, ultrafiltration, or immuno-capture) and characterisation through a minimum of two orthogonal modalities, such as TEM or cryo-EM, particle sizing (NTA/DLS/IFC), and immunomarkers (e.g., CD9/CD63/TSG101/ALIX). We prioritised research that elucidated seminal heterogeneity by density (e.g., EV-H/EV-M/EV-L) or size and that expressly concentrated on NV co-isolation. To maintain clinical relevance, the selected papers documented either functional endpoints in sperm (including motility metrics, CASA outputs, capacitation markers, acrosome status, zona binding, ROS/DFI, DNA damage repair surrogates, Ca^2+^ signalling, or hyperactivation) or clinical outcomes (such as fertilisation rate, 2PN formation, blastulation, pregnancy or live birth, and TESE success in NOA/OA). Human data were prioritised, while animal studies were selectively included when they clarified mechanisms not accessible in humans (e.g., epididymal EV cargo transfer to sperm, small-RNA remodelling during transit, recipient-site tropism on the head/neck/flagellum, EV-mediated Ca^2+^ handling, redox buffering, and immune modulation). Investigations of testicular or epididymal tissue were deemed valid if they demonstrated EV-to-sperm transfer or characterised region-specific EV characteristics with possible translational relevance.

We excluded records that mentioned “exosomes” or “microvesicles” solely as pellets without EV validation, reports that relied exclusively on precipitation (e.g., PEG) without subsequent purification or orthogonal characterisation, and studies that conflated EVs with lipoproteins or protein aggregates. We excluded works lacking a sperm/seminal axis (such as female-tract EV studies devoid of sperm interactions), studies on semen chemistry unrelated to EVs, conference abstracts lacking comprehensive data, texts not in English, narrative pieces without primary evidence, and duplicate analyses of the same cohort that presented no novel methods or endpoints. We omitted or assigned lesser significance to trials that failed to control for or report leukocytospermia, abstinence interval, fever/infection, medicines, or varicocele status, as these variables could alter EV profiles or sperm functionality. For biomarker assertions, we required explicit definitions of the cases (e.g., OAT, asthenozoospermia, NOA versus OA), predetermined objectives, and appropriate controls. For mechanistic in vitro studies, it was necessary to delineate the dosage, time, and origin (e.g., spEV, epididymosome, or prostasome), conduct fundamental quality assurance of the preparation, and, when feasible, identify the recipient. We utilised reviews and methodological papers for snowball sampling; however, we did not consider them as evidence until they presented new data and re-analyses.

### 2.3. Data Extraction and Integration

We employed a standardised template to obtain the biofluid or tissue source (semen/spEVs, epididymal fluid/epididymosomes (mouse), prostatic secretions/prostasomes, testicular preparations), the isolation and cleanup workflow (dUC, DG, SEC, ultrafiltration, immuno-capture), and the characterisation level (TEM or cryo-EM, particle sizing via NTA/DLS/IFC, immunomarkers) from each qualifying record we extracted. Authors meticulously categorised semen into fractions, documenting the specific labels and conditions for each fraction (e.g., EV-H/EV-M/EV-L density windows, NV removal, and buffer systems). For functional analysis, we documented the dosage and exposure duration of EVs, the localisation of the recipient on the sperm cell (head/neck/flagellum when feasible), the assay conditions (capacitation media, pH, progesterone challenge), and the primary endpoints (CASA motility parameters, capacitation markers and tyrosine phosphorylation, CatSper/PMCA4 readouts, acrosome status, zona binding, ROS/DFI, DNA damage). We compiled phenotypic definitions (OAT, asthenozoospermia, NOA vs. OA), inclusion criteria, and ART outcomes (fertilisation rate/2PN, blastulation, pregnancy/live birth, TESE success) for clinical studies. In cargo investigations, we identified the analyte type (proteins, miRNAs, tRFs, metabolites, lipids), the impacted pathways, and the direction of change relative to controls.

To enhance comparability, we standardised units (e.g., EV dosage by particle number or protein mass per 10^6^ sperm; ROS/DFI scales), modified summary statistics when feasible, and indicated that whether outcomes were normalised to sperm counts or motile fractions. Multi-arm tests were summarised per arm, preserving comparators within the study. When cohorts appeared to intersect (same centre/authors/timeframe), we eliminated duplicates by retaining the most comprehensive or methodologically rigorous report and cross-referencing the others in documentation.

The synthesis advanced throughout three dimensions. Initially, from an origin-centric perspective, testis-derived vesicles, epididymosomes, prostasomes, and composite small extracellular vesicles (spEVs) were discussed individually to acknowledge tract biology. Secondly, function-centric: maturation, capacitation signalling, motility/hyperactivation, acrosome reaction/zona interaction, oxidative stress and DNA integrity, and immunological crosstalk were addressed as distinct modules, facilitating the alignment of EV fraction biology with pathway-specific outcomes. Third, phenotype-centric: human data were categorised by clinical classification (OAT, asthenozoospermia, NOA/OA, and TESE prediction, recurrent implantation failure contexts), with mechanistic animal studies integrated to clarify causality (e.g., EV-mediated small-RNA remodelling during epididymal transit).

This is a narrative review; we did not perform a meta-analysis of effect sizes; rather, we provide the direction and approximate magnitude of effects when relevant (e.g., percentage change in progressive motility, fold-change in phosphorylation markers, area-under-curve for biomarker models). We allocated weights to the evidence according to: (i) prioritisation of human data over animal data for clinical endpoints; (ii) adherence to MISEV-aligned isolation and characterisation; (iii) resolution of seminal heterogeneity via fractionation and NV control; (iv) sample size and replication; and (v) consensus among independent laboratories. An in-text study of conflicting data and possible sources of discordance (definitions of fractions, precipitation versus gradient methods, leukocytospermia, abstinence duration, specimen management, progesterone challenge protocols) was performed.

We categorised each proposed biomarker according to its intended application (diagnostic versus prognostic), specimen type (whole semen, spEVs, or fraction), assay method (proteomic panel, miRNA/tRF signature, or metabolite/lipid feature), endpoint (e.g., TESE success in NOA, fertilisation/blastulation), and validation status (internal cross-validation or external cohort), while documenting calibration and decision thresholds when available. We reviewed the dosage. The method and timing of preparing sperm (density-gradient centrifugation [DG] or swim-up), compatibility with IVF/ICSI protocols, and any safety signals (microbial/endotoxin control, off-target effects) for translational interventions such as EV add-back, modified EVs, and cryoprotection.

### 2.4. Assessment of Quality

Two tiers of methodological excellence were assessed: EV-specific rigour and narrative synthesis rigour. We followed the SANRA-style domains for the narrative dimension, encompassing clear rationale, adequate search and selection, appropriate referencing, scientific reasoning, and transparency regarding constraints. We allocated weights to research according to their alignment with MISEV and established single-particle analytics for the EV dimension. We specifically favoured studies that (i) examined NV co-isolation and addressed seminal heterogeneity through density or size classification (e.g., EV-H/EV-M/EV-L), (ii) detailed pre-analytical variables (abstinence duration, liquefaction period, centrifugation and viscosity management, storage conditions/freeze-thaw cycles), (iii) correlated fraction identity with function in relation to dosage, exposure duration, and recipient-site tropism on sperm (head/neck/flagellum), and (iv) employed established isolation methodologies (dUC, DG, SEC, ultrafiltration, or immuno-capture) alongside orthogonal characterisation techniques (TEM/cryo-EM and particle sizing via NTA/DLS/IFC, as well as immunomarkers such as CD9/CD63/TSG101/ALIX).

Functional studies that incorporated replicates, appropriate blinding, positive and negative controls, and quantitative endpoints pertinent to sperm biology (e.g., CASA motility metrics, capacitation markers/tyrosine phosphorylation, Ca^2+^ measurements such as CatSper/PMCA4 activity, acrosome status, zona binding, ROS/DFI) were awarded higher grades. The omics articles were evaluated based on sample size, precise case definitions (OAT, asthenozoospermia, NOA vs. OA), batch control, multiple-testing correction, and validation by internal cross-validation and, preferably, external cohorts. Extra credit was awarded to clinical studies for its repeatability across centres, covariate adjustment (age, abstinence, leukocytospermia, varicocele, fever/infection, medicines), and pre-defined endpoints (fertilisation rate/2PN, blastulation, pregnancy/live birth, TESE success).

The narrative summary of bias risk across studies identified recurring threats, including precipitation-only isolates lacking cleanup or orthogonal characterisation, confusion between EVs and lipoproteins/protein aggregates, absence of negative controls, undefined or mixed fractions labelled as “exosomes,” small, single-centre cohorts, and inconsistent capacitation/progesterone challenge protocols. We examined fractionation protocols, particle dosing (per 10^6^ sperm or per mL), storage and thawing methods, and the presence of leukocytes or microorganisms in instances where the results were ambiguous. In the absence of human resolution, animal data were utilised as a supplementary mechanism, whereas human data were prioritised for clinical inference.

To ensure certainty, we employed a three-tiered strength-of-evidence rubric during synthesis: preliminary (small or methodologically limited studies, precipitation-only isolates, or endpoints distant from clinical utility), moderate (human data fulfilling most criteria or animal data with definitive mechanistic insights and translational plausibility), and strong (human data adhering to MISEV-aligned methodologies, sufficient sample size, orthogonal characterisation, consistent functional or clinical endpoints, and at least one independent replication). Prior to composing the Results section, the author team conducted a thorough verification of quality assessments and research classifications, resolving any discrepancies through consensus.

### 2.5. Methods and the Correlation Between Dimensions and Origin for Differentiating Seminal Extracellular Vesicles

The populations of EVs present in semen originate from the prostate (“prostasomes”), the epididymis (“epididymosomes”), sperm, and, post-ejaculation, the female reproductive tract with which sperm interact. The similarity in size of these groups precludes size as a determinant of their origin. Reliable differentiation requires the simultaneous use of purpose-specific separation, orthogonal single-particle characterisation, uniform pre-analytics, and origin-enrichment markers. In pre-analytical handling, it is crucial to monitor leukocytospermia, liquefaction duration, viscosity control, abstinence period, and the interval between ejaculation and processing. It should specify if plain semen, seminal plasma, or sperm–EV co-incubations were examined. Laboratories often use a combination of differential ultracentrifugation and iodixanol DG to isolate and fractionate vesicles by buoyant density while eliminating NV nanoparticles such as lipoproteins and protein/RNA complexes. SEC may further reduce NV carryover, and when more resolution is required, immuno-capture or asymmetric flow fAF4 may be used. High-sensitivity flow cytometry, or imaging flow cytometry, facilitates particle-level phenotyping. Following the characterisation of an individual particle, transmission electron microscopy or cryo-electron microscopy can ascertain the morphology of the membrane-bound particle, while nanoparticle tracking analysis, dynamic light scattering, or tuneable resistive pulse sensing can quantify its dimensions and concentration. The origin is determined by analysing patterns of enrichment rather than relying on a single marker. Prostate secretory proteins and complement regulators are typically present in prostatosome-rich fractions alongside tetraspanins. Εpididymosome-enriched fractions contain epididymal surface/cargo proteins and small RNAs delivered to sperm (microRNAs and tRNA-derived small RNAs), sperm-derived vesicles are enriched with sperm-membrane proteins from the head/acrosome or flagellum, female-tract extracellular vesicles (oviduct/uterus) encountered post-ejaculation can be identified by tract proteins (e.g., OVGP1) and cargos transferred to sperm (e.g., PMCA4). Conversely, NV markers like as ApoA1/ApoB and several soluble proteins suggest non-vesicular transport. Employing dose-defined co-incubations (particles per 10^6 sperm, timed exposure) alongside suitable controls (NV-enriched fractions and heat-inactivated EVs) and evaluations of capacitation, Ca^2+^ handling, acrosome reaction, reactive oxygen species, and mitochondrial membrane potential (ROS/Δψm), or zona binding, functionally associates these assignments with biological processes. Origins should be established based on size, density, marker panels, and function, with any inferential mapping explicitly articulated. The conventional size bands (small EV ~30–150 nm, microvesicle-like ~100–1000 nm, apoptotic bodies ≥ 500 nm) may vary according on preparation methods and may be located in diverse environments. To ensure reproducibility of results, reports must encompass pre-analytics, the precise separation methodology and collected fractions, orthogonal characterisation (morphology, sizing, markers), the NV-depletion strategy, the EV dosage/exposure utilised in functional assays, and a comprehensive rationale for the origin assignment, detailing the densities, markers, and functional evidence that substantiated the determination.

### 2.6. Limitations

It is crucial to recognise the many limitations inherent in this narrative evaluation. Initially, significant disparities persist in the methodologies employed for isolation, pre-analytical conditions, density-fractionation protocols, and the differentiation between co-isolated NVs and authentic EVs in the existing data regarding sperm-derived and seminal EVs. These methodological shortcomings hinder cross-study comparisons and may obfuscate the functional importance of certain EV subpopulations. Secondly, most mechanistic understanding is derived from animal models that may not correctly represent human reproductive physiology, particularly in studies examining EV-sperm docking dynamics, CatSper/PMCA4 regulation, or epididymosome-mediated short RNA transfer. Third, the findings lack broad applicability due to the predominance of small, single-centre human research that seldom use external validation or standardised clinical objectives like as fertilisation rate, blastulation, euploidy, or live birth. Fourth, achieving repeatability across labs is challenging due to the lack of agreement on reference standards for particle dosage, extracellular vesicle measurement, and functional assay calibration. This research integrates clinical and mechanistic data; nevertheless, it cannot eradicate publication bias or misidentified studies, since it is a narrative review rather than a systematic one. These constraints highlight the need for multicentre validation, standardised techniques, and clinically relevant EV phenotyping in future research initiatives.

## 3. Origins and Biogenesis of Male Reproductive Tract Extracellular Vesicles

According to our research semen is not a homogeneous extracellular vesicle mixture. It comprises vesicles released at many locations along the male reproductive tract, including the testis, epididymis, and accessory glands, in addition to those released by the spermatozoa themselves. Each source provides a distinct membrane architecture and cargo logic (proteins, lipids, short RNAs), interacting with sperm at different stages of their life cycle, from post-testicular maturation to ejaculation and transit within the female reproductive tract. Comprehending this source-function map is essential for analysing molecular data and employing extracellular vesicles clinically as biomarkers or functional modulators.

### 3.1. Extracellular Vesicles Derived from the Testis

The testis operates as an EV-rich signalling centre, enabling vesicular transport between tubules and the interstitium, crossing the blood-testis barrier in both directions, and linking tubular gametogenesis to interstitial endocrine control [35]. In our review, extracellular vesicles generated from stem cells serve as a molecular rheostat for the niche. They convey specific miRNA and protein cargo to SSCs to promote their survival and differentiation, while simultaneously providing regulatory signals to LCs that influence steroidogenesis [36]. An important example is the transfer of miR-145-5p from SC extracellular vesicles to LCs, which suppresses SF-1 and reduces the steroidogenic program (STAR, CYP11A1, HSD3B). This renders the interstitium endocrinologically inactive despite the consistent presence of LCs [37]. Further evidence suggests that other SC-EV miRNAs, including miR-486-5p, are involved in IGF/AKT-related targets, leading to reduced steroidogenesis under heightened stress conditions. Simultaneously, SC EVs provide pro-survival signals, including CCL20, that inhibit LC apoptosis and enhance interstitial function, suggesting that the same vesicle source can engage in bidirectional regulation based on the context [38]. In conjunction with SC signals, the immunological and circulatory systems have a role: TM exosomes alleviate radiation-induced harm to spermatogonia, whereas TEC-derived EVs have proteome/miRNA patterns that are thought to affect spermatogenic pathways, thus linking endothelium status to germline viability. This evidence demonstrates that testicular EV trafficking is a vital component of soma-germline communication, which preserves SSC maintenance, regulates proliferation checkpoints, and aligns maturation with endocrine support [39].

The similar circuitry enables the recognition of paths to pathology. When SC EV production is diminished quantitatively or altered qualitatively, SSCs forfeit trophic support and proliferation regulation, whilst LCs undergo transcriptional repression of steroidogenesis. This interaction undermines epididymal conditioning and ultimately impairs sperm function, despite seemingly normal semen parameters [40]. Conversely, an abundance of inhibitory payloads from SC EVs may render tissues hypogonadal by silencing SF-1 targets, without any discernible loss of histological Leydig cells. Testicular EVs penetrate the BTB and enter the luminal flow, allowing their signatures to be detected in semen. Exosomal miR-210-3p has been identified as a potential biomarker for Sertoli cell injury in varicocele [41]. This demonstrates that testis-derived substances can function as a liquid biopsy for intratesticular disorders, facilitating azoospermia classification, preparation for TESE, and toxicant evaluation. Region-specific investigations of the male reproductive system highlight that the origin of EVs affects distinct tetraspanin and cargo phenotypes; therefore, accurate identification of “testicular” signals in ejaculate requires fractionation and comprehensive EV characterisation to avoid conflation with subsequent epididymal or prostatic vesicles [42]. The molecular data substantiate the incorporation of testis-EV analytics into male-factor protocols: they disclose upstream lesions in niche signalling and endocrine interactions, provide non-invasive biomarkers of SC/LC stress, and underscore actionable targets for reinstating steroid support and germline homeostasis prior to sperm entering the epididymis.

### 3.2. Epididymosomes

Epididymosomes constitute the principal post-testicular extracellular vesicle system that alters the sperm surface, proteome, lipidome, and small RNA composition throughout their passage from the caput to the cauda. They derive from epididymal epithelium and engage with sperm in a region- and domain-specific manner, involving transient docking and fusion that selectively targets the head, equatorial segment, neck, or principal piece, contingent upon the segment of origin and the prevailing tetraspanin/adhesion milieu. Proteomic analyses in mammals reveal a conserved toolkit comprising CRISP family members, ADAM metalloproteases, MIF, PDIA3, CLU, MFGE8, CD63/CD9 complexes, c-SRC, TCP1/CCT chaperonins, metabolic enzymes, and redox systems, whose enrichment corresponds with essential functions for motility, zona interaction, redox regulation, capacitation, and acrosome functionality. Samples of human epididymal fluid and vas deferens demonstrate maturation-related alterations in sperm-associated proteins, including ADAM7, AKAP3, GAPDHS, LDHC, TEKT3, and CRISP1. Evidence indicates that ADAM7 and CRISP1 facilitate gain-of-function transfers to the plasma membrane, while CD9 and certain adhesion signals are selectively removed from specific head microdomains. Lipidomics and electron microscopy reveal a simultaneous elevation in sphingomyelin and cholesterol concentrations. This leads to the formation of detergent-resistant microdomains that organise ion channels, fusogens, and zona-binding complexes in advance [43]. This persistent, raft-rich state is primed for the rapid cholesterol efflux and membrane re-fluidisation that occurs during capacitation. Fluctuations in vesicular pH, androgen sensitivity, and luminal constituents such CRISP cofactors, β-defensins, and epididymal lipocalins, which are affected by segment, seem to modify the final composition and fusogenicity of epididymosomes. This establishes a molecular basis for the gradual addition and removal of surface characteristics that standard sperm counts or morphology cannot disclose [44].

The transfer of small RNA is essential for epididymosomes, directly connecting the physiology of the tract to paternal epigenetic provisioning. miRNAs in mice and tsRNAs are the two types of small RNAs that are most common in epididymal extracellular vesicles. Their cargo changes depending on the epithelial baseline (the cellular content outside of vesicles) and the different parts of the epididymis (caput, corpus, and cauda). These patterns are not consistent with passive leakage. Instead, they are consistent with ceramide-dependent budding of small EVs (neutral-sphingomyelinase-driven membrane remodelling) and selective loading via RNA-binding adaptor proteins (proteins that recognise specific RNA motifs or modifications) [45]. Studies including mice, cattle, and humans demonstrate that families such as let-7, miR-26/103/191/200, and tRF-Gly-GCC/tRF-Glu-CTC are prevalent in epididymosome cargo, and their selective transfer to maturing sperm alters the small-RNA composition between the caput and cauda. The experimental modification of epididymal extracellular vesicle small RNAs affects zygotic gene expression and early cleavage dynamics, thereby connecting vesicle-associated RNAs to embryo competence. Environmental and metabolic signals such as nutrition, temperature stress, and endocrine disruptors modulate the epididymal epithelial transcriptome and, subsequently, the extracellular vesicle RNA profile, establishing a biological mechanism by which paternal exposures affect the gamete. This RNA channel is both dynamic and reactive to exposure. Men exhibiting similar normal semen characteristics may have differing results in fertilisation and blastocyst development, since seminal extracellular vesicles may affect capacitation, redox equilibrium, and Ca^2+^ regulation by providing functional proteins to sperm [46].

Multivalent tetraspanin lattices and adhesive ligands that concentrate in certain sperm areas promote short-range lipid mixing or endocytosis, hence facilitating epididymosome–sperm interactions. Connections between MFGE8 and integrins, contacts between annexin and phosphatidylserine, and platforms formed by CD9/CD63 enhance the interaction; nevertheless, local curvature and cholesterol concentrations affect the probability of fusion and the degree of cargo deposition [47]. Thus, domain specificity arises: zona-binding proteins, ADAMs, and fusogens are localised to the apical head and equatorial segment, while axonemal regulators, glycolytic enzymes, and redox buffers are concentrated in the midpiece and principal piece, affecting ATP supply, reactive oxygen species regulation, and flagellar waveform control [48]. In vitro transfer assays employing purified vesicles exhibit swift transfer of proteins and RNAs, alongside an increase in fertilisation-associated markers tyrosine phosphorylation patterns, hyperactivation potential, and acrosome readiness even in the absence of the complete complexity of the epididymal lumen [49]. These findings corroborate a theory wherein recurrent, microdomain-specific interactions during transit gradually create the molecular apparatus necessary for capacitation and gamete interaction.

Pathology is intrinsically connected to this system and provides significant explanatory capacity for “idiopathic” male-factor anomalies. Systemic stressors that modify the epididymal epithelium such as obesity, hypoxia linked to varicocele, febrile illness, smoking, and urogenital infection redirect vesicle cargo from antioxidant enzymes, chaperonins, and zona-interaction proteins to inflammatory mediators. Asthenozoospermia, premature capacitation, aberrant or spontaneous acrosome reaction, and elevated sperm DNA fragmentation are linked to a dysregulated maturation environment marked by oxidative stress and impaired vesicle-mediated cholesterol/Ca^2+^ buffering, despite sperm count and morphology being within reference ranges [50]. The ineffectiveness of targeted elimination processes is detrimental: the retention of immature head proteins can hinder receptor clustering, disrupt the organisation of CatSper and PMCA4, and desynchronise the capacitation clock, leading to insufficient zona binding or dysregulated acrosome exocytosis during insemination [51]. A quantitative decrease in epididymosome-derived tsRNAs and miRNAs disrupts paternal RNA provision, correlating with abnormal first-cleavage kinetics and premature developmental arrest in model systems, thereby establishing a mechanistic connection between tract inflammation and embryonic stage failures observed in clinical contexts [52]. These pathways align with the intersections in the human proteome between semen extracellular vesicles and epididymosome databases, as well as with segment-specific short RNA analyses that link altered profiles to motility, apoptosis, and DNA repair mechanisms [53].

Epididymosome signals in semen act as potential non-invasive indicators of upstream dysfunction; nevertheless, they require careful separation from various seminal extracellular vesicle populations and non-vesicular nanoparticles. Proteins and small RNAs displaying segment bias in spEV fractions may differentiate caput and cauda defects, aid in distinguishing OA from NOA contributions to the luminal pool, and improve TESE planning by indicating whether intratesticular production or post-testicular maturation is the limiting factor [53]. In ART processes, characterising epididymosome contents may aid in selecting sperm preparation methods, predicting sperm response to capacitation stimuli, and identifying candidates for adjuvant EV “add-back” to restore absent maturation signals before insemination or ICSI. Engineered vesicles that replicate epididymosome cargo antioxidant modules, zona-binding proteins, or specific small RNA mixtures represent a rational progression, provided that production complies with efficacy and safety standards, and exposure is appropriately timed to avert premature acrosome destabilisation [54]. Analytics centred on epididymosomes can detect tract abnormalities overlooked by traditional semen examination, utilising standardised isolation, fractionation, and single-particle phenotyping. This strategy facilitates targeted techniques to enhance fertilisation potential and embryo development in a therapeutically relevant manner.

### 3.3. Supplementary Accessory-Gland Extracellular Vesicles, Including Prostasomes

Following a review of the structure and principal indicators of prostasomes, we concentrate on the timing of activities associated with fractions, including lipid transfer, immunological regulation, and Ca^2+^ licensing. Prostasomes, the primary accessory-gland extracellular vesicles in semen, along with epididymal vesicles, constitute a highly intricate small extracellular vesicle pool characterised by diverse biochemical and biophysical properties [55]. High-resolution iodixanol gradients segregate seminal extracellular vesicles into consistent density subtypes (EV-H, EV-M, and EV-L) while isolating non-vesicular components. This indicates the presence of various marker constellations (CD9/CD63/TSG101/ANXA1) and distinct morphologies, such as the “small tubule” category, which is abundant in EV-L and observable via cryo-EM. Datasets at the proteomic level (>4200 proteins) indicate that pathways are categorised by subtypes [56]. Markers such as PSCA, ANXA1, and GLIPR2 can elucidate the accessory-gland origin of specific populations, indicating that the ejaculate derives from sources beyond the epididymis. This architecture has a functional purpose beyond just aesthetics. The density of vesicles and their surface phenotype can indicate the cargo delivered to sperm, its docking location, and the molecular modules it modulates in the minutes following ejaculation. This establishes a communication system governed by the prostate and divided by segments [57].

The fast, pH-regulated provision of Ca^2+^ machinery to ejaculated sperm, a typical function of prostasomes, facilitates progesterone-induced hyperactivation and efficient flagellar flipping. Proteases are positioned upstream of CatSper gating, PMCA4 efflux, and the tyrosine phosphorylation cascades involved with capacitation, facilitated by fusion at mildly acidic pH and the direct transfer of calcium signalling components, including cADPR/CD38-linked elements [58]. Prostasomes can initiate or inhibit the acrosome response, contingent upon the dosage, timing, and fraction. This induces exocytosis upon contact with the zona, rather than occurring prematurely upon release. Molecular pathology refers to the study of disease at the molecular level. Standard semen analysis fails to provide mechanistic insights into idiopathic motility abnormalities and unsuccessful IVF inseminations [59]. This encompasses the inability to fuse prostasomes and sperm, depletion of Ca^2+^ regulators, or an inclination towards inhibitory glycoproteins (such as glycodelin in severe asthenozoospermia), resulting in diminished progesterone responsiveness, disruption of capacitation timing, and impaired hyperactivation despite normal sperm counts.

These molecular elements coalesce to generate therapeutic and biomarker opportunities. Research identifying unique PSCA/ANXA1/GLIPR2-positive extracellular vesicle populations highlights the importance of their source and the potential for non-invasive collection from semen [60]. Comparative investigations reveal that prostasome-associated proteins and RNAs are modified in non-normozoospermic males, whereas density-resolved spEV proteomes identify panels capable of differentiating functional phenotypes and tracking glandular contributions. In a clinical context, if preparations adhere to GMP standards and are scheduled to prevent premature acrosome destabilisation, a prostasome-deficient or EV-M-deficient profile necessitates the use of particular fractions or modified EVs to re-establish Ca^2+^ signalling and antioxidant functionality during sperm preparation [61]. Analysing upstream secretion patterns by accessory-gland EV analytics might improve OA/NOA assessments in azoospermia, streamline diagnostic processes, and customise ART treatments when fertilisation failure is associated with a prostasome/immune or redox profile instead of solely a motility shortage.

In the female reproductive tract, sperm-derived and seminal extracellular vesicles significantly influence immunological modulation, fostering an environment conducive to sperm viability and early embryonic development. Prostasomes, a primary category of extracellular vesicles, include complement regulating proteins such as CD46, CD55, and CD59. These proteins inhibit the production of C3/C5 convertase and save sperm against degradation by complement. These EVs possess ligands for NK-cell receptors, such as CD48, which inhibit NK-cells from cytotoxic activity and prevent sperm from exiting the uterus. Seminal extracellular vesicles promote a tolerogenic phenotype marked by reduced antigen presentation and increased IL-10 production, while suppressing pro-inflammatory cytokine release from dendritic cells and macrophages. Extracellular vesicle miRNAs significantly influence immune-regulatory pathways. For instance, miR-223, miR-146a, and miR-148a inhibit TLR signalling and NF-κB activation, hence reducing inflammation in the uterus after semen deposition. In vitro studies further indicate that spEVs reduce collateral damage to sperm membranes by preventing the development of neutrophil extracellular traps. These systems collaborate to establish an immune-privileged milieu by regulating innate cells such as neutrophils, macrophages, and NK cells, while promoting the proliferation of regulatory T-cells, essential for successful fertilisation and implantation.

### 3.4. Semen Heterogeneity (Dimensions, Density, Origin, and Function)

Seminal extracellular vesicles comprise a complex system of subpopulations characterised by varying sizes, densities, membrane structures, cargo contents, and biological activity. Density gradients reliably partition spEVs into EV-H, EV-M, and EV-L, while concurrently removing NV, subsequent cryo-EM analysis reveals distinct morphologies within these classifications, including cup-shaped small EVs enriched for CD9/CD63/TSG101 and an EV-L subset distinguished by “small tubules” with lipid-rich walls [62]. Proteomics and lipidomics reveal that pathways are divided into fractions: EV-M possesses significant antioxidant mechanisms, including GSTM2 and peroxiredoxins. EV-L contains many lipids that facilitate the formation of rafts and scaffolds, which are believed to assist in membrane priming. EV-H frequently has an abundance of prostasomes and possesses components for managing Ca^2+^ and fusogens [63]. This structure influences sperm biology: EV-H and NV enhance hyperactivation and capacitation markers, EV-M reduces intrinsic ROS and safeguards DNA, while EV-L appears to stabilise head-neck membranes prior to acrosome readiness [64].

Molecular pathology delineates alterations in these fractions and their contents. Despite normal numbers and shape, asthenozoospermia frequently presents issues with prostasome-sperm fusion or a deficiency of EV-H prostasomal Ca^2+^ modules, resulting in diminished progesterone responsiveness and ineffective flagellar switching [65]. When EV-L increases but EV-M remains unchanged, membranes may appear “stable” yet fail to facilitate timely cholesterol efflux, resulting in delayed or dysregulated capacitation. A high DFI or recurrent inadequate blastulation is associated with the depletion of EV-M antioxidant buffering, rendering chromatin more susceptible to strand breakage and protamine oxidation induced by ROS. Infection and inflammation introduce an additional dimension: Leukocytospermia delivers extracellular vesicles and proteases from white blood cells that can destroy zona-interaction proteins and modify the sperm extracellular vesicle proteome. Conversely, chronic prostatitis induces a shift in prostasomal cargo from Ca^2+^ components to inflammatory glycoproteins, a pattern that correlates with unexplained infertility following IVF insemination [66]. Varicocele and hypogonadism exemplify endocrine and vascular stressors that induce fraction imbalances undetectable by standard semen analysis. These imbalances arise from altered source contributions, including reduced prostasome load and disordered epididymosome signatures [67].

The recipients possess varying requirements. Variations in sperm maturation, membrane organisation, and receptor accessibility within a single ejaculate generate uptake-biased subpopulations. Midpiece-targeted vesicles that transport metabolic enzymes and redox buffers preferentially associate with cells exhibiting elevated Δψm, whereas head-focused vesicles containing zona-binding proteins and fusogens preferentially attach to sperm with intact apical rafts [68]. Swim-up and DGC alter this environment in distinct manners: DGC diminishes NV contamination; nevertheless, it may also eliminate loosely attached vesicles and alter the apparent fraction potency in functional assays. Swim-up enhances the quantity of cells responsive to EV-H Ca^2+^ payloads, although it may also diminish the requisite number of EV-M connections necessary for regulating ROS [69]. If unregulated and unreported, these pre-analytical factors liquefaction duration, viscosity management, centrifugation parameters, freeze-thaw cycles, and abstinence period can lead to contradictory results among centres and alter the composition of EVs and the capacity of sperm to uptake them.

Table 1 illustrates the progression of semen extracellular vesicles from “origin” to “function.” Molecular markers are linked to particular functional deficits: epididymosomes are associated with maturation and zona interaction, prostasomes with Ca^2+^ licensing and immunological modulation, EV-M with redox ceiling/Δψm stability, and seminal-vesicle EVs with viscosity/raft environment.

### 3.5. Prospective Developments

The main aim of future research should be to convert new molecular discoveries into clinically proven tools and treatment methods. A primary objective is to ensure that EV isolation, fractionation, and single-particle characterisation protocols are consistent and adhere to MISEV recommendations for applicability across various locations. Comprehensive multicentre clinical investigations are essential to correlate distinct extracellular vesicle subpopulations. Specifically, prostasomes, epididymosomes, and density-defined seminal extracellular vesicle fractions (EV-H/EV-M/EV-L) with fertilisation, blastulation, embryo viability, and live birth outcomes. Mechanistic studies should explain how the integration of molecular pathways, including CatSper–PMCA4 signalling, epididymosome-derived small-RNA remodelling, and redox buffering, governs capacitation time, acrosome stability, DNA integrity, and oocyte activation via EV cargo. Developing liquid biopsy signatures derived from EVs for the stratification of azoospermia and the prediction of TESE, as well as investigating engineered EVs as targeted adjuvants to address absent maturation signals or safeguard sperm during cryopreservation, constitute further objectives. Accomplishing these goals would close the existing gap between clinical andrology and molecular extracellular vesicle biology, enabling more personalised, mechanism-based approaches for assisted reproduction and male infertility. Currently, there is no experimental evidence to substantiate the notion that sperm-derived or seminal extracellular vesicles selectively interact with X- or Y-bearing spermatozoa. Recent studies suggest that sperm maturation stage, membrane microdomain composition, capacitation state, and epididymal location of origin are more significant factors for EV docking and cargo transfer than chromosomal identity. Consequently, any potential EV-related distinctions between X and Y sperm remain hypothetical and have yet to be empirically validated in either animal models or human subjects.

## 4. Molecular Cargo of Sperm-Relevant EVs

### 4.1. Proteome

The EV proteome is modular and encoded by its source, and it is applied to sperm with remarkable spatial precision. Epididymosomes frequently transport a collection of maturation tools present in human epididymal fluid and in other animals. Members of the CRISP family (e.g., CRISP1/4) regulate the timing of capacitation and zonal interaction, ADAMs (notably ADAM7) facilitate secondary binding and membrane remodelling. SPAM1/PH-20 assist in penetrating the cumulus matrix. MIF is involved in motility and redox control. PDIA oxidoreductases modify disulphide bonds on surface receptors. CLU acts as an extracellular chaperone that stabilises client proteins in an oxidative environment [70]. MFGE8 links vesicle integrins to sperm membranes for efficient docking. TCP1/CCT chaperonins aid in the folding of actin/tubulin and the organisation of the peri-axonemal cytoskeleton [71]. AKAPs (AKAP3/4) anchor PKA near CatSper/flagellar machinery and glycolytic and redox enzymes (GAPDHS, LDHC, ENO, PRDXs, SOD, and GST isoforms) enhance ATP supply and reactive oxygen species buffering in the midpiece and principal piece. These cargos pertain to many checkpoints, encompassing bioenergetics (GAPDHS/LDHC + AKAP-localised signalling), chaperoning (CLU/TCP1-CCT), membrane fusion competence (tetraspanin-organised receptor clusters + PDIA-dependent disulphide switching), and zona recognition (CRISP/ADAM/SPAM1). Transfer is specific to domains: principal-piece cargos alter dynein functionality and waveform transitions, midpiece cargos enhance mitochondrial performance and mitigate ROS to safeguard mtDNA and axoneme, while head-centric proteins aggregate at the acrosomal cap and equatorial segment to form receptor-fusogen complexes. Pathology results from incomplete or inadequately timed sequences. Inadequate PDIA/CLU support causes misfolding and proteolysis of surface receptors, leading to premature capacitation or unsuccessful zona binding despite normal morphology [72]. The absence of head-bound CRISP/ADAM/SPAM1 modules leads to inadequate zona adhesion and unstable acrosome condition. The reduction in AKAP-anchored signalling and glycolytic enzymes results in diminished hyperactivation despite normal numbers. The missing information is in the proteome supplied by epididymal extracellular vesicles, rather than in the triad of count, motility, and shape. This clarifies the coexistence of asthenozoospermia, increased DFI, or fertilisation failure with “normal” semen parameters [73].

Prostasomes introduce an additional, rapid-acting proteome that alters signalling within minutes of ejaculation. The cADPR/CD38 axis components, which are regulators of Ca^2+^ handling that interact with CatSper and PMCA4, lipid-active enzymes facilitating cholesterol efflux and bilayer fluidisation, and dense tetraspanin webs (CD9/CD63/CD81) that cluster receptors at the neck and midpiece, represent a subset of the numerous Ca^2+^ tools and scaffolds enabling the progesterone response in EV-H-enriched prostasomal fractions [74]. Prostasomes also provide proteases and glycoproteins that alter the acrosome’s readiness. The actions of these regimes are contingent upon the dosage and fraction, which induces exocytosis when the zona interacts with the acrosome rather than prematurely. Fractionation elucidates function. EV-M contains numerous antioxidant enzymes, namely GSTM2 and peroxiredoxins, which reduce intrinsic sperm reactive oxygen species, safeguard chromatin crosslinks, and stabilise DNA. EV-L is rich in lipids and appears to facilitate the organisation of head-neck membranes, which is essential for the prompt response of the acrosome and the conduction of the neckpiece [75]. EV-H, frequently abundant in prostasomes, promotes capacitation markers and hyperactivation while reinstating the kinetics of progesterone-induced tyrosine phosphorylation. Our data are shown in Table 2.

### 4.2. Minor RNAs and Epigenetic Signals

During fertilisation, sperm-derived small RNAs (miRNAs, tsRNAs) from the epididymis are integrated into the egg, resulting in a temporary excess of testis-enriched small RNAs in the zygote compared to maternal and somatic levels [52]. The cargo comprises a selectively curated library abundant in let-7, miR-26/103/191/200 families, along with several 5′ tRNA segments (tRF-Gly-GCC, tRF-Glu-CTC, tRF-Val, among others). It does not reflect a mirror image of the epithelial transcriptome. RNA-binding adaptors and ceramide/ESCRT-associated pathways are utilised in sorting. Chemical markers such as m^5C/m^1A on tRFs, m^6A on certain miRNAs, and 2′-O-methylation at the termini of piRNAs contribute to stability in the extracellular environment and facilitate Argonaute loading post-uptake [76]. Domain-specific delivery aggregates regulatory RNAs at the sperm’s head and periacrosomal cytosol, allowing them to exist as ribonucleoprotein complexes. These short RNAs regulate the gene programs of blastomeres and the initial cleavage of the embryo prior to the commencement of zygotic transcription. A causal relationship exists between vesicle RNA and embryo competence, since the removal or dilution of epididymal EV RNAs alters the expression of genes associated with metabolism, cell cycle, and chromatin remodelling, hence disrupting the kinetics of the initial cell cycle [77]. Reintroducing these traits restores them. Concurrently, miRNAs encapsulated in vesicles specifically target sperm pathways in situ, including mitochondrial redox regulation, apoptotic checkpoints, and CatSper/PMCA4 scaffolding. This presents a biochemical mechanism via which post-testicular cues modify acrosome readiness, motility, and the timing of capacitation, without affecting sperm numbers or morphology [78].

Pathology follows exposure biology. Heat stress, hypoxia (varicocele), obesity, smoking, illness, and endocrine disruptors alter the RNA-sorting machinery and the epithelium of the epididymis [79]. Consequently, the vesicle library shifts towards inflammatory and stress-response miRNAs, while distancing itself from developmental and redox-protective tsRNAs. Clinical signs include asthenozoospermia, increased DFI, premature or impaired capacitation, and recurring insufficient blastulation despite “normal” semen tests. This results in a sperm small-RNA landscape that promotes apoptosis, reactive oxygen species generation, and inadequate calcium handling. The absence of specific paternal tRF sets diminishes zygotic metabolism and cleavage symmetry. This establishes a mechanistic link between embryonic developmental failure and tract inflammation. These alterations are encapsulated in vesicles, facilitating access to upstream pathology via a genuine liquid biopsy. This remains accurate even when the cellular RNA production is minimal [80].

The prostate and seminal vesicles contribute additional layers to the endometrium and sperm post-ejaculation through the utilisation of spEV small RNAs. Prostasome-rich fractions supply miRNAs that preserve the peri-conception immunological milieu by reducing complement damage and leukocyte activation [81]. Target/pathway studies reveal enrichment in the PI3K–AKT, cAMP–PKA, and MAPK/ERK signalling cascades that govern capacitation. Moreover, oviductal and seminal-plasma EVs convey miRNAs to sperm; specifically, OEV-derived miR-34c-5p localises to the centrosome, whilst SPEV-derived miR-222 is taken up by sperm. In humans, prostasomes maintain the acrosome by regulating signalling events and preventing capacitation and spontaneous acrosome reactions; acrosomal instability during insemination may arise from the removal or imbalance of this input [82].

Clinical translation is already feasible. SpEV miRNA panels classify asthenozoospermia according to redox or signalling abnormalities, distinguish non-obstructive azoospermia from obstructive azoospermia, and predict the effectiveness of testicular sperm extraction [83]. In IVF/ICSI, integrated miRNA–tRF signatures correlate with fertilisation rates and blastulation, exceeding conventional semen parameters and conforming to fractional biology (EV-H/Ca^2+^ axis versus EV-M/redox axis). Long non-coding RNAs and circular RNAs present in small extracellular vesicles contribute to the biomarker landscape [84]. They may also provide information regarding the gland of origin, aiding in the differentiation between prostatic and epididymal diseases. To implement this, reliable pre-analytics, calibrated qPCR/NGS pipelines with spike-in controls and external standards, and fraction-aware sampling (eliminating NV while keeping EV-H/M/L identification) are required. Upon standardisation, small-RNA readouts can inform interventions such as timing AOA when RNA indicators suggest ineffective Ca^2+^ licensing, administering antioxidants or anti-inflammatory agents when EV-M signatures are diminished, or reintroducing specific vesicle preparations or engineered EVs containing corrective miRNA/tRF cocktails to restore maturation cues prior to ICSI or insemination [85]. Table 3 shows a summary of important small-RNA panels, the directions of phenotypic changes, and how they can be used in medicine.

### 4.3. Lipids and Metabolome

EV membranes serve as active platforms with a distinct composition that provide biophysical order, signalling competence, and metabolic resilience to sperm throughout late epididymal transit and ejaculation. Epididymosomes are abundant in SM, cholesterol, and various glycerophospholipids (PC/PE/PS/PI) characterised by segment-specific fatty-acyl chains, with a higher concentration of PUFA, including DHA, in the cauda. They possess PS patches that are acknowledged by annexins and tetraspanin lattices (CD9/CD63/CD81) [86]. This lipid composition stabilizes the outer acrosomal membrane prior to capacitation by augmenting the liquid-ordered raft area, reducing baseline permeability, and inhibiting stochastic Ca^2+^ leakage across the head and neck bilayer. The lipid-active enzymes (SMase2/acid SMase, sPLA_2_, PLC/PLD isoforms, LPCATs) and scaffold proteins that organise PIP_2_/PIP_3_ microdomains collaborate to facilitate hemifusion, enhance local curvature (via ceramide/lysophospholipids), and alter the arrangement of receptor/fusogen clusters (ADAMs/IZUMO1–JUNO axis) at the apical head [87]. The transport of SM/cholesterol and gangliosides from vesicles to sperm occurs simultaneously. Raft remodelling occurs concurrently with phosphorylation alterations that prepare CatSper for placement and PMCA4 for activation, with lipid transfers associated with AKAP3/4 and sAC–cAMP–PKA signalling domains [88]. The glycolytic transfers (GAPDHS/LDHC and substrates) facilitate the maintenance of ATP microdomains along the principal segment. The metabolic buffers supplied by epididymosomes polyamines (spermine/spermidine), carnitine/carnitine esters, lactate/pyruvate, citrate and antioxidant enzymes (SOD1, PRDXs, GPX4) enhance the GSH/GSSG ratio, maintain Δψm stability, and regulate axonemal dynein stability [89]. Subsequent to ejaculation, prostasome-rich EV-H fractions in mouse conclude this process by dispatching cholesterol-transfer proteins and lipases that collaborate with albumin and bicarbonate to regulate cholesterol efflux, facilitate local membrane fluidisation, and remodel CatSper-proximal rafts. They also include CD38/cADPR and other Ca^2+^-licensing components that convert the progesterone pulse into robust intracellular Ca^2+^ oscillations and timely hyperactivation [90]. EV-L isolates raft-forming lipids and curvature scaffolds that appear to prepare the head–neck interface for stable docking and timely acrosome activation; EV-M fractions focus on redox regulation, being enriched with GSTM2, PRDX5/6, and thioredoxin-associated modules that mitigate basal ROS and protect PUFA-rich membranes from peroxidation. The vesicle network functions in two phases: the post-ejaculation licensing phase, during which female-tract vesicles and seminal vesicles (prostasomes/seminal EVs) regulate cholesterol efflux and calcium handling (e.g., PMCA4 transfer); and the epididymal stabilisation phase, wherein lipids, proteins, and small RNAs are conveyed by epididymal vesicles to maintain membrane integrity, suppress calcium/reactive oxygen species signalling, and keep the acrosome inactive.

Molecular disease with identifiable clinical manifestations arises when any element of this lipid-metabolite pathway is deficient, altered, or mismatched. The depletion of EV-M antioxidant activity eliminates the GST/PRDX inhibition on intrinsic ROS, resulting in raft fragmentation, lipid peroxidation (4-HNE/MDA adducts), and CatSper desensitisation [91]. Despite normal counts, nuclear and protamine crosslinks exhibit oxidative stress, DFI increases, Δψm fluctuates, and progressive motility deteriorates. A specimen biased towards EV-L lipids, without compensating EV-M support, results in uncertain fertilisation while exhibiting normal morphology. It also impedes the passage of cholesterol, dissociates the acrosome’s readiness from contact with the zona, and excessively stabilises the head-neck membrane [92]. Depletion of prostasome-rich EV-H or ineffective fusion results in sperm lacking Ca^2+^ licensing and neckpiece scaffolds, hence reducing the progesterone response and altering tyrosine-phosphorylation kinetics. Individuals with this syndrome demonstrate asthenozoospermia or unexplained IVF fertilisation failure despite standard semen analysis [93]. Infection and inflammation alter vesicle lipids bidirectionally: Leukocytospermia introduces neutrophil-derived oxidants and enzymes, particularly PMN-elastase and MPO, into semen. These are associated with elevated amounts of reactive oxygen species and membrane damage. Oxidative and proteolytic stress may disrupt the tetraspanin and annexin-organised platforms that facilitate interactions between extracellular vesicles and sperm, as well as sperm-head lipid rafts. This may complicate vesicle docking and increase the likelihood of acrosomal dysregulation (an early or atypical acrosome response) [94]. Prostatitis and dysbiosis increase oxidised glycolipids and decrease complement-control lipids in prostasomes, disrupting pH-regulated fusion and reducing the duration of viable capacitation [95]. The metabolic syndrome disturbs the balance of vesicular ceramide and diacylglycerol, causing sperm to preferentially engage in PKC-biased signalling that misaligns the time of capacitation. Hypoxia associated with varicocele induces the preferential distribution of epididymal lipids towards shorter, oxidation-sensitive acyl chains and decreases the levels of polyamines. This diminishes Δψm resilience and elevates midpiece ROS levels [96]. Methodological artefacts may replicate sickness. The absence of NV clearance or EV-H/M/L fractionation masks obscures whether kind of protection redox, Ca^2+^ licensing, or membrane priming is really lacking. Precipitation-only isolates co-concentrate NV lipoproteins (ApoA1-rich HDL), which indiscriminately absorb cholesterol and provoke premature capacitation [97]. Fraction aware lipidomics/metabolomics, coupled with focused functions such as ROS quenching by EV-M, progesterone rescue by EV-H, and cholesterol efflux kinetics, elucidate the primary lesion and enable actionable therapies. When EV-L levels are elevated, incorporate add-backs that stabilise membranes while maintaining redox equilibrium; when progesterone responses are suboptimal, restore prostasome delivery pathways or implement timed EV-H supplementation; additionally, modify sperm preparation techniques (distinguishing between swim-up and DGC, avoiding PEG precipitation, and regulating freeze-thaw cycles) to preserve optimal EV interactions while mitigating NV-induced artefacts [98]. The lipid and metabolite signatures encoded by these vesicles transform an undetectable membrane disorder into a manageable target. This enables ART to be customised in manners that transcend the count–motility–morphology framework. Table 4 delineates the lipid and metabolite attributes for each fraction, including their biophysical roles, indicators of illness, and potential remedies.

### 4.4. Targeting Mechanisms and Surface Ligands

A consortium of tetraspanins, integrins, annexins, glycoconjugates, and charged lipids forms an “address label” that instructs the vesicle surface on how to identify extracellular vesicles and sperm. This marking indicates the docking location, the pathway of absorption, and the depth of cargo delivery. Tetraspanin webs (CD9/CD63/CD81) construct nanoscale platforms on epididymosomes that assemble adhesion molecules, partner receptors, and MFGE8 (RGD–integrin bridge). These webs further link to PS-rich regions that attract annexins, establishing robust connections at the equatorial segment and acrosomal cap, and glycans enhance specificity [99]. Sperm lectin-like receptors only recognise vesicle sialylation and fucosylation patterns, whereas heparan-sulphate-binding motifs direct vesicles to head microdomains containing zona-binding complexes. Charge and curvature are significant. Lysolipids and ceramide enhance negative curvature and reduce the energy barrier for complete fusion [100]. Elevated cholesterol and sphingomyelin form organised rafts that facilitate hemifusion. Protonation establishes the foundation. Prostasomes exhibit excellent binding and fusion capacity at the acidic endpoint of the ejaculate and cervical mucus to ensure that docking, fusion, and signalling transpire in a precise temporal sequence. As pH and bicarbonate concentrations rise above capacitation thresholds, they subsequently facilitate the transmission of Ca^2+^-licensing processes [101].

Origin imprints are designed to focus on the logic associated with sperm domains and their roles. Caput epididymosomes preferentially target the head and equatorial segment, since they deposit zona-interaction proteins, chaperones, and disulphide isomerases that enhance receptor architecture and position the acrosome [102]. Cauda vesicles migrate towards the primary piece and neckpiece, transporting redox enzymes, AKAP-scaffolded kinases, and axonemal regulators near the mitochondria and CatSper domains. Prostasome-rich EV-H preferentially adorns the neck/midpiece, where tetraspanin–integrin lattices intersect with PMCA4 and lipid-active enzymes. This converts the progesterone pulse into a robust Ca^2+^ response and hyperactivation. Oviductal and uterine vesicles form a reciprocal layer inside the female reproductive system. They transmit PMCA4, SPAM1, and specific miRNAs to the sperm, enhancing Ca^2+^ homeostasis and cumulus penetration at the appropriate time and location. These patterns emerge from multivalent interactions with avidity beyond that of any singular receptor-ligand combination, rather than from arbitrary adhesion [103]. Modifying the glycan code or tetraspanin ratio might reduce the likelihood of fusion or misdirect vesicles to incorrect domains, all while maintaining overall binding consistency in an inconspicuous manner.

The interface often serves as the origin of the issue. Inflammation, infection, and metabolic stress alter the glycosylation and tetraspanin ratios on source cells. This results in vesicles with altered avidity or tropism that are misdirected [104]. Prostatitis reduces pH-sensitive fusion at the neckpiece and lowers progesterone-induced Ca^2+^ transients via modifying prostasomal surfaces to pro-inflammatory glycoforms and decreasing complement-regulatory expression. This manifests clinically as insufficient hyperactivation and failure of IVF fertilisation despite appropriate sperm numbers [105]. Hypoxia coupled with varicocele is a molecular mechanism that results in unsuccessful zona adhesion despite normal morphology. It alters glycosylation patterns in the epididymis and reduces MFGE8 on vesicles, complicating head-targeted docking and resulting in undeveloped zona-binding assemblies [106]. Leukocytospermia, characterised by the injection of CD45^+^ white blood cell-derived extracellular vesicles (WBC-EVs) containing proteases that cleave tetraspanin partners (such as ADAM10/17-mediated shedding) and myeloperoxidase oxidants that compromise phosphatidylserine/annexin interfaces, results in diminished vesicle docking, premature acrosome instability, and erratic capacitation timing [107]. Non-volatile contaminants complicate targeting further: HDL particles rich in ApoA1 compete for cholesterol, compromising the raft’s structure, which results in premature capacitation and diminishes the likelihood of successful EV fusion [108]. Despite vesicle binding, uptake may be unsuccessful due to the influence of pH and bicarbonate on the kinetics of actin remodelling and receptor internalisation [109]. If the semen is not adequately buffered during preparation or handling, it alters the endocytic pathway, resulting in cargo being trapped at the membrane. This results in diminished zona responses and flat tyrosine-phosphorylation trajectories that a conventional semen analysis cannot anticipate [110].

These surface codes may be used for therapeutic intervention. Employing imaging-flow or nano-flow cytometry with panels for CD9/CD63/CD81, MFGE8/integrins, PS/annexins, PSCA/GLIPR2 (accessory-gland origin), and CD45 (WBC-EV contamination), fraction-aware phenotyping of spEVs identifies the disrupted interface: head docking (zona/fusion axis), neck/midpiece licensing (Ca^2+^/hyperactivation axis), or global adhesion avidity (glycan/tetraspanin axis). When EV-M antioxidant indicators are available but head-targeting ligands are deficient, restoration requires re-establishing epididymal-style docking (MFGE8/tetraspanin ratios) instead of using generic antioxidants [111]. In males with many failed IVF attempts, a prostasome-lean EV-H signature and low MFGE8 may forecast suboptimal progesterone responses. This indicates that including EV-H or modified vesicles containing PMCA4/CD38 modules at the optimal moment during sperm production is advantageous. In contrast, leukocyte reduction and anti-inflammatory treatment are beneficial before any vesicle-based intervention in samples showing increased CD45^+^ EVs and reduced tetraspanin intensity. Upon elucidating the pathophysiology from the vesicle surface, a formerly imperceptible adhesion/fusion defect emerges as a focal point for diagnostics and tailored ART protocols. This occurs far in advance of its manifestation in fertilisation results.

## 5. Functional Roles in Sperm Physiology

### 5.1. Mature Post-Testicular

Iterative interactions with epididymosomes that modify, remove, and rearrange membrane proteins, lipids, and small RNAs in a segment-specific order are essential for post-testicular maturation in the epididymis. Sperm exiting the testis are transcriptionally inactive and functionally deficient [21]. Tetraspanin lattices, MFGE8-integrin bridges, and PS-annexin interfaces enable docking on designated head, equatorial, neck, and principal-piece microdomains. Hemifusion or endocytosis then moves cargo with submicron accuracy. The head receives zona-interaction and fusion elements (CRISP, ADAMs, and SPAM1) that keep the receptor structure stable in an oxidising lumen; AKAP-scaffolded kinases, glycolytic enzymes, and redox buffers that coordinate cAMP/PKA signalling with mitochondrial output and ROS thresholds; and the main piece is tuned for waveform switching by the delivery of axonemal regulators and membrane organizers that set up CatSper fields and PMCA4 access [112]. Ceramide/lyso-lipid transfer changes the curvature and asymmetry of the leaflets to make it easier for fusion events to happen later. Lipid exchange, on the other hand, stops random Ca^2+^ leaks and stops the acrosome from becoming unstable too soon by increasing the SM/cholesterol content and expanding the ordered raft area [113]. The second axis of maturation is epigenetic: vesicle-borne miRNAs and tRFs replace the testis-biased small-RNA set in a caput → cauda gradient, creating a library that survives ejaculation and can change the first cell cycles of the embryo. They also change sperm-intrinsic pathways (PI3K–AKT, MAPK, Nrf2) that control when capacitation, motility, and acrosome readiness happen, but they do not change the number or shape of the sperm [114]. So, maturation is a program that is timed and encoded in vesicles, not just a single switch. It entails the sequential deposition of protein, lipid, and RNA layers onto the same cell, ensuring that capacitation competence manifests exclusively at the cauda stage.

Pathology appears whenever the program loses accuracy in dose, timing, or targeting. Even when the concentration and morphology are fine, heat and low oxygen levels in varicocele or febrile illness stop the epididymal antioxidant and chaperone cargo from working and shift small-RNA sorting towards stress modules [115]. This causes asthenozoospermia with a high DFI; low-grade infections and leukocytospermia inject proteases and MPO-derived oxidants, which break apart tetraspanin partners and oxidise head rafts. This makes it less likely that vesicles will fuse and makes it more likely that the acrosome will become unstable too soon. Smoking, metabolic syndrome, and endocrine disruptors alter epithelial glycosylation and tetraspanin ratios, diminishing MFGE8-mediated head docking and resulting in underdeveloped zona-binding assemblies [116]. Obstruction alters the luminal environment: Prolonged stasis alters the pH, viscosity, and protein composition of the epididymis, impairing vesicle biogenesis and fusogenicity, resulting in inadequately edited ejaculated sperm even after patency is restored [102]. Some of the molecular signs of these lesions are lower PDIA/CLU support with misfolded surface receptors and unstable capacitation trajectories; truncated small-RNA hand-offs with impaired zygotic kinetics despite seemingly normal semen reports; loss of head-bound CRISP/ADAM/SPAM1 with poor zona adhesion and irregular acrosome timing; and depletion of AKAP-anchored glycolytic and kinase sets with weak hyperactivation and flat progesterone responses. Bulk “exosome” measurements hide the main lesion because each defect is linked to a fraction. Fraction-aware readouts show whether maturation failed at lipid priming, protein transfer, or RNA provisioning. They also explain why men with the same semen profiles diverge at fertilisation and blastulation [117].

This biology has direct effects on how it can be used in medicine. If the output from the testicles is fine but the editing after the testicles is not, you can use proteins and small RNAs that are biased towards segments and can be recovered from spEV fractions to separate OA from NOA contributions to the luminal signature, index caput versus cauda failure, and plan TESE [118]. A maturation signature characterised by low head-targeting ligands and depleted redox modules in ART workflows necessitates modifications in preparation to preserve beneficial EV interactions, alterations in the timing of progesterone exposure, and considerations regarding the reintroduction of specific fractions or engineered EVs that restore antioxidant capacity, AKAP-scaffolded signalling, or absent head receptors without inducing premature capacitation [119]. Men who have repeated failures after ICSI can find out if the problem is with oocyte activation, licensing of Ca^2+^ responses, or upstream editing by combining EV-based maturation profiling with PLCζ status and capacitation tests. This changes a phenotype that was previously “idiopathic” into a plan that is based on a mechanism and can be managed.

### 5.2. Capacitation Signalling

Capacitation is the systematic process of altering the membrane and transmitting signals that prepares sperm to adhere to the zona and initiate an acrosome response at the appropriate location. Post-ejaculation, the dilution in tract fluids elevates intracellular pH, bicarbonate, and calcium concentrations. This initiates protein tyrosine phosphorylation, soluble adenylyl cyclase activity, and cAMP/PKA signalling. Lipid reorganisation and regulated cholesterol outflow both enhance membrane fluidity. CatSper-mediated calcium influx and meticulously regulated reactive oxygen species formation facilitate hyperactivation and acrosome competency. Microvesicles, such as female reproductive tract and seminal extracellular vesicles, modify some stages of these processes. Seminal microvesicles (from prostasome and sperm) transport cholesterol and sphingomyelin, decapacitation signals, complement regulators, and antioxidant enzymes to stabilise head membrane domains, inhibit premature Ca^2+^ influx and reactive oxygen species, and maintain acrosome quiescence during initial phases. Female reproductive tract microvesicles (oviductal/uterine extracellular vesicles) facilitate the organisation of tetraspanin-based docking platforms, provide small RNAs and regulatory factors that modulate kinase pathways (cAMP/PKA, PI3K–AKT, MAPK/ERK) governing capacitation kinetics, and transfer proteins and lipids that assist in cholesterol management and the maintenance of stable Ca^2+^ levels (for instance, PMCA-type Ca^2+^ extrusion). This two-stage input initial stabilisation from seminal microvesicles followed by activation from female-tract microvesicles facilitates the coordination of tyrosine phosphorylation, pH/Ca^2+^ dynamics, cholesterol efflux, and redox balance, ensuring that hyperactivation and the acrosome reaction occur at the appropriate moment near the oocyte, neither prematurely nor belatedly.

A stable epididymal state is transformed into a fertilisation-competent phenotype by a controlled reprogramming of the sperm plasma membrane, ion fluxes, and kinase-phosphatase networks, a process known as capacitation [120]. The trigger set is external to the body: peri-ejaculatory signals from extracellular vesicles interact with alterations in the bicarbonate, albumin, and potassium/pH levels of the female reproductive tract. A-kinase anchoring proteins (AKAP3/4), protein phosphatases (PP1/PP2A), and Src-family kinases modulate the phosphorylation of specific head and flagellar proteins that serve as capacitation indicators following the entry of HCO_3_^−^ into the cell via electrogenic NBC cotransporters and anion exchangers, subsequently activating sAC (ADCY10) and elevating cAMP and PKA activity [121]. NHEs and Hv1 extrude H^+^ ions from the cell, altering the pH gradient from the head to the main piece, while albumin simultaneously extracts cholesterol from raft microdomains. This reduces the cholesterol-to-phospholipid ratio, hence enhancing membrane fluidity. On this scaffold, the CatSper domains of the major segment become accessible to progesterone. ABHD2 enhances this response by preventing 2-AG from obstructing the channel, whereas PMCA4-mediated Ca^2+^ efflux alters the oscillatory patterns [122]. Prostasome-rich EV-H fractions provide CD38/cADPR modules and lipid-active enzymes that alter the configuration of local microdomains surrounding CatSper/PMCA4, facilitating the attachment to the neckpiece and midpiece and accelerating this licensing process within minutes after ejaculation. Residual modifications in the epididymis (receptor aggregation and PDIA-mediated disulphide switching) inhibit acrosome motility until zona binding occurs. The cumulative result constitutes a phase transition: head receptors gain competence but remain constrained, membrane order and cAMP–PKA signalling intensify concurrently, and the flagellum transitions to a hyperactivated waveform only upon reaching certain amounts of progesterone and HCO_3_^−^ [123].

Pathology occurs when any component of this cascade operates ineffectively or becomes disorganised. Genetic or pharmacological inhibition of sAC (ADCY10) results in sperm that seem normal but are unable to capacitate promptly, diminish the cAMP spike, and obstruct PKA-dependent tyrosine phosphorylation [124]. Despite normal numbers and morphology, the loss-of-function of CatSper (channel subunits, auxiliary proteins, or improper membrane composition) inhibits progesterone-induced Ca^2+^ influx, hence preventing hyperactivation and zona penetration. Less severe lesions, such as abnormalities in the ABHD2 pathway or cholesterol-enriched microdomains that impede efflux, result in diminished Ca^2+^ transients and erratic fertilisation during IVF [125]. PMCA4 loss or mislocalisation prolongs intra-flagellar Ca^2+^ plateaus, disrupting waveform regulation and depleting ATP microdomains. Oxidation of PUFA-rich membranes and lipids next to CatSper diminishes channel sensitivity and reduces the hyperactivation window. This is often due to insufficient EV-M antioxidant cargo. Oxidative stress also crosslinks protamines, increasing DFI and dissociating embryo competence from capacitation [126]. The acrosome may fail to exocytose or properly bind to the zona when the tail is hyperactivated, as the head receptors are inadequately formed owing to deficiencies in the epididymis, including insufficient transport of CRISP/ADAM/SPAM1 and defective chaperoning by PDIA/CLU. The Ca^2+^-licensing “boost” is diminished downstream due to a deficiency of prostasomes or fusion failure, which often occurs in prostatitis and dysbiosis. This results in diminished fertilisation rates after insemination and insufficient progesterone responses in vitro, which cannot be predicted by a conventional semen study.

In this physiology, extracellular vesicles play a crucial role in determining whether capacitation occurs positively or negatively. EV-M regulates baseline reactive oxygen species and maintains the integrity of the raft, facilitating optimal albumin-mediated cholesterol efflux. EV-L provides the curvature of the head-neck interface and raft scaffolds that maintain stability until full contact is achieved. EV-H activates the Ca^2+^ pathway and connects progesterone detection with CatSper domains [127]. The phenotype alters when the equilibrium is disrupted: Reduced EV-H results in progesterone insensitivity and flat trajectories of tyrosine phosphorylation; diminished EV-M induces early lipid peroxidation, attenuated or premature phosphorylation, an elevation in DFI, and a reduction in blastulation; while elevated EV-L in the absence of EV-M leads to excessively stable membranes, delayed cholesterol efflux, and acrosome readiness that is incongruent with zona binding [128]. The introduction of leukocytospermia and protease-rich white blood cell extracellular vesicles results in the cleavage of tetraspanin partners, oxidation of phosphatidylserine-annexin interactions, a reduction in vesicle docking, and inconsistent, noisy capacitation. Precipitation techniques that co-enrich NV lipoproteins indiscriminately extract cholesterol and induce premature capacitation [94]. If HCO_3_^−^/albumin or pH levels are inadequately regulated during preparation, the sAC pulse is attenuated and endocytosis of EV payload is altered [129]. Pathology may arise even during laboratory processing.

These mechanical maps have a direct influence on the bench. Fraction-aware tests differentiate a Ca^2+^-licensing defect from a redox impairment or a membrane-priming mistake by connecting tyrosine phosphorylation kinetics, progesterone-induced Ca^2+^ profiles, and hyperactivation with a patient’s extracellular vesicle signature. Subsequently, biology directs the interventions: when progesterone responses are stable, reinstate EV-H input or administer engineered vesicles containing PMCA4/CD38; when phosphorylation is erratic and DFI is elevated, augment EV-M or provide targeted antioxidants; and when acrosome timing is inconsistent, sustain EV-L interactions while safeguarding against premature cholesterol efflux. Capacitation readouts and extracellular vesicle profiling aid in evaluating the need for artificial oocyte activation during intracytoplasmic sperm injection or the possibility of upstream conditioning to restore function when the sAC/CatSper pathways are inherently impaired. Capacitation represents the convergence of membrane biophysics, ion channels, and vesicle biology, facilitating fertilisation or leading to the “idiopathic” failures seen by physicians. By analysing and modifying the EV-modulated steps, that line transitions from descriptive to actionable.

### 5.3. Motility and Hyperactivation

Progressive motility is a novel characteristic of the axoneme, peri-axonemal structures, and a membrane-signalling sheath that is continually modified by extracellular vesicles from the prostate and epididymis. Epididymosomes construct the essential scaffolding required for phosphorylation cycles to occur with spatial accuracy [130]. These scaffolds include AKAP3/AKAP4 to maintain sAC–cAMP–PKA nanodomains throughout the principal segment, GAPDHS/LDHC to sustain glycolysis inside the fibrous sheath, and chaperones/oxidoreductases to ensure the appropriate folding of dyneins and outer dense fibre proteins in an oxidative lumen [131]. These vesicles serve as membrane organisers that aggregate PMCA4 and CatSper fields into microdomains capable of rapid Ca^2+^ uptake and release, while also enhancing CABYR, PRKAR2A/B, and calcineurin regulators that govern beat-to-beat dephosphorylation. The interchange of ceramide and lyso-lipid alters the curvature and asymmetry of the leaflets, facilitating hemifusion and maintaining vesicle docking throughout transit [132]. The transfer of lipids enlarges the raft area and enhances the rigidity of the head-neck bilayer, so safeguarding the midpiece electrical milieu and diminishing background Ca^2+^ leakage that might disrupt beat patterns. These modifications provide the foundation for the ejaculatory ‘license’ by associating progesterone detection with CatSper activation and PMCA4-mediated clearance. Prostasome-rich extracellular vesicles dock at the neckpiece and midpiece, delivering calcium tools (lipid enzymes that modulate local fluidity, CD38/cADPR modules) while enhancing intracellular pH regulation via Hv1/NHE scaffolding and promoting bicarbonate accessibility to sAC hotspots. AKAP-tethered PKA induces episodes of tyrosine phosphorylation and alters the conformation of actin around the axoneme to facilitate flagellar movement through cervical mucus and cumulus [133]. The flagellum transitions from low-amplitude progressive waves to high-amplitude, asymmetric hyperactivation alone upon reaching certain thresholds of bicarbonate, pH, and progesterone.

Hyperactivation is a Ca^2+^ patterned waveform that is meticulously regulated by metabolic processes and temporal factors, rather than just increased motility. PKA-primed phosphorylation cascades and calcineurin (PPP3CC)-dependent dephosphorylation modulate dynein activity on a cycle-by-cycle basis; PMCA4 and mitochondrial Ca^2+^ uniporter dynamics restore basal Ca^2+^ levels to avert tetanic arrest and ATP depletion and CatSper fields produce bursts of Ca^2+^ that propagate from the principal piece to the midpiece [134]. Electric vehicles regulate the extent of their operational range by modulating the flagellum’s capacity to manage ions and its resilience to redox stress. The progesterone-induced Ca^2+^ surge is superficial; tyrosine-phosphorylation pathways stabilize, AKAP microdomains shift, and hyperactivation remains subdued despite normal cellular counts and morphology when EV-H input is deficient or fusion is ineffective (e.g., prostatitis, dysbiosis) [135]. In response to progesterone stimulation, CASA subsequently exhibits reduced VSL/VAP and diminished ALH. Excessive EV-L lipids coupled with a scarcity of antioxidant EV-M lipids results in too organised and oxidised membranes. Raft fragmentation desensitises CatSper, leads to the accumulation of 4-HNE/MDA adducts on dynein chains and outer dense fibres, increases internal friction, and results in the cell exhibiting “stop-go” trajectories with a shallow zona approach and inadequate mucus penetration [74]. Conversely, an unaltered EV-H/M/L balance enhances the dynamic range facilitating swift acceleration into hyperactivation, regulated decay back to progressive motion, and effective navigation through the cumulus matrix due to brief yet pronounced Ca^2+^ bursts, synchronised phosphorylation cycles, and the preservation of lipid microdomains for sufficient duration to enable productive activity [88].

Energetics and redox reactions establish the parameters of signalling, whereas vesicles are also calibrated to these constraints. Glycolysis energises the distal primary piece, whereas oxidative phosphorylation supplies ATP to the midpiece [136]. Epididymosomes provide GAPDHS/LDHC, aldolase/ENO partners, and substrates or substrate shuttles to maintain ATP microdomains precisely where dynein need them. They also maintain the stability of mitochondrial cristae function by regulating redox processes [137]. To maintain baseline reactive oxygen species within the “signalling, not damaging” range, EV-M incorporates GSTM2, peroxiredoxins, thioredoxin modules, and the capacity to regenerate glutathione. In the absence of this brake, Δψm fluctuates, the leak at complex I/III increases, and lipid peroxidation disrupts both CatSper gating and axonemal sliding. This results in CASA profiles exhibiting reduced progressive motility, an irregular BCF, and an elevated DFI downstream [138]. The phenotype deteriorates with an increase in lesions: Leukocytospermia reduces the hyperactivation period by introducing WBC-EV proteases that cleave tetraspanin partners used by EVs for docking, together with myeloperoxidase oxidants that damage principal-piece lipids, despite seemingly normal baseline motility [139]. Endocrine and metabolic stress (hypogonadism, metabolic syndrome) modifies epididymal lipid sorting and small-molecule cargo, leading to impaired pH_i control (NHE/NBC coupling) and sAC activation, which therefore reduces the cAMP spike required for PKA to commence beat switching. Prolonged spins or extended stays at ambient temperature degrade the vesicle signals that inform extracellular vesicles about their mobility under in vitro fertilisation settings. This occurs due to the removal of weakly attached EVs or the oxidation of raft lipids. Precipitation techniques that co-enrich NV lipoproteins indiscriminately aggregate cholesterol and result in premature capacitation [140].

This biology may provide targeted modifications at the bench, rather than using generic “antioxidants” or implementing arbitrary alterations to the medium. Utilising live Ca^2+^ imaging and tyrosine-phosphorylation kinetics, fraction-aware EV phenotyping (EV-H/Ca^2+^ licensing, EV-M/redox control, and EV-L/membrane priming) can more accurately forecast the efficacy of progesterone addition and elucidate the discrepancies in CASA results compared to mere count-motility assessments. The strategic administration of EV-H, or engineered vesicles containing PMCA4/CD38 modules, can reinstate the CatSper–PMCA loop in specimens with diminished hyperactivation and superficial Ca^2+^ transients, without triggering premature acrosome loss; the augmentation of EV-M, or the introduction of targeted antioxidant cargo restores waveform stability, preserves Δψm, and improves blastulation in samples marked by DFI and midpiece ROS [141]. Preserving EV-L connections while regulating raft integrity via regulated albumin/bicarbonate exposure inhibits premature capacitation and restores zona-synchronised exocytosis in conditions marked by heightened membrane order and delayed cholesterol efflux. Seemingly technical decisions, such as swim-up vs. DGC, buffer pH/HCO_3_^−^ regulation, and the avoidance of PEG precipitation, systematically alter EV-sperm interactions and hence affect the motility phenotype [142]. The output of a vesicle-encoded program mostly involves motility and hyperactivation. Utilising fraction aware assays to analyse this signature and progressively refining it, an “idiopathic” motility issue may be transformed into a controllable, mechanism-based remedy that integrates molecular biology with clinical results.

### 5.4. Acrosome Reaction and Zona Pellucida Interaction

The AR is a precisely regulated exocytotic process that discharges a combination of hydrolytic enzymes and unmasking ligands to facilitate secondary binding and penetration through the ZP. Prior to zona contact, EVs regulate the timing and preparedness of the sperm head [143]. Throughout epididymal transit, epididymosomes deliver head-specific cargos such as CRISP family proteins (e.g., CRISP1), ADAMs (e.g., ADAM7), SPAM1/PH-20, PDIA oxidoreductases, CLU/TCP1 chaperones, and tetraspanin-scaffolded adhesion complexes. These cargos alter the configuration of the apical plasma membrane, the outer acrosomal membrane, and the equatorial segment [144]. These proteins establish fusogens (partners of IZUMO1) and receptor clusters for first zona pellucida contact (complexes recognising ZP3/ZP4). They assist in preserving conformations in an oxidative environment by facilitating and modifying disulphides. Lipid transfer from epididymosomes generates curvature fields that facilitate hemifusion with arriving vesicles, inhibit premature acrosomal vesiculation, eliminate unstable head glycoproteins, and enlarge sphingomyelin/cholesterol rafts [145]. Post-ejaculation, EV-H fractions rich in prostasomes adhere to the neck/head contact, transmitting lipid mediators and Ca^2+^-licensing enzymes that align AR competence with capacitation. The acrosome remains dormant until zona ligation provides the ultimate impetus [146]. Subsequently, PMCA4 clearance and PKA-primed tyrosine phosphorylation facilitate the propagation of CatSper-mediated Ca^2+^ bursts to the head via reorganised microdomains. The procedure has two primary components: “licensing” ejaculation and “priming” the epididymis. The first segment positions zona-binding ligands and fusogens, enhances the resilience of membrane microdomains against noise, and links Ca^2+^ dynamics with a brief interval during which AR is advantageous rather than detrimental [147].

Molecular disease arises when any component of this EV-encoded program is absent, misdirected, or improperly timed. When the levels of head-targeting epididymosome cargo are diminished (low CRISP/ADAM/SPAM1; insufficient PDIA/CLU support), receptor fields become sparse, misfolded, or improperly grouped [148]. This weakens initial ZP binding, prevents the maturation of the equatorial segment, and diminishes the efficiency of IZUMO1–JUNO interaction post-AR. When prostasome input is obstructed, as seen in prostatitis or dysbiosis, the Ca^2+^ “licensing” enhancement at the neckpiece is absent. Despite normal numbers and morphology, hemizona failure occurs due to the absence of AR when the zona is ligated or when it occurs just partly. CatSper transients have low amplitude, PMCA4 recycling occurs at a sluggish pace, and phosphorylation pathways stabilize. Premature AR is an additional failure mechanism often seen in specimens exhibiting inflammation or significant neovascularisation [149]. Head rafts disintegrate, NV lipoproteins indiscriminately extract cholesterol, leukocyte-derived oxidants and proteases sever tetraspanin partners and oxidise PS/annexin interfaces, while acrosomes release en masse during capacitation or swim-up, resulting in “ghosts” that are incapable of secondary binding. The depletion of EV-M antioxidant buffering permits the peroxidation of PUFA-rich head membranes and the carbonylation of zona-interactive proteins simultaneously, therefore diminishing CatSper sensitivity at the head and compromising the integrity of the outer acrosomal membrane [150]. This is clinically seen as a high DFI, atypical FITC-PSA patterns, and suboptimal fertilisation after IVF insemination, despite adequate progressive motility. Redox pathology has equal significance. The phenotype is marked by normal morphology, diminished zona adhesion, and delayed or incorrectly timed acrosome reaction. Hypoxia and systemic metabolic stress associated with varicocele induce alterations in head glycan codes (sialylation/fucosylation) on sperm and vesicles. This reduces the likelihood of ZP receptors binding to them and redirects EV docking away from the apical cap.

The EV targeting logic elucidates the locations and mechanisms of AR occurrence. Cauda-biased vesicles provide adhesion modulators and PS-annexin machinery, stabilising the equatorial segment as the post-AR fusion location [151]. Caput-biased vesicles preferentially target the apical head and equatorial segment, depositing zona-interaction ligands, fusogens, and disulphide isomerases essential for the correct fusion of membranes. To prevent tetanic plateaus in the head, prostasomes Favor the neck/midpiece–head junction, where tetraspanin–integrin lattices converge with PMCA4 and lipid enzymes to regulate the influx and efflux of Ca^2+^ inside the cell [152]. When this topography is disrupted, for instance, by altered tetraspanin ratios on vesicles, reduced MFGE8, or receptor loss on sperm, cargo delivery is misdirected to an incorrect domain or ceases at the membrane. AR becomes spatially unfocused, exhibiting patchy vesiculation that diminishes IZUMO1 exposure and reduces fusion competency. Even seemingly innocuous actions in the laboratory might reveal underlying disease [153]. For instance, prolonged exposure of raft lipids to room temperature can result in oxidation; precipitation-based extracellular vesicle preparations may co-enrich non-viable components while depleting functional EV-H/M fractions, and excessive exposure to bicarbonate or progesterone can induce premature cholesterol efflux and off-target phosphorylation, resulting in early androgen receptor activation prior to zona contact [154].

These mechanistic maps serve as the foundation for diagnoses and therapies. Fraction-aware spEV phenotyping and functional assessment (live Ca^2+^ imaging at the apex, hemizona tests, IZUMO1 exposure, progesterone- or zona-induced AR with FITC-PSA/lectin labelling) Differentiate between premature AR (NV-high; oxidised rafts; depleted EV-M), ligand/scaffold deficit (low epididymosome head cargo; poor ZP binding), and licensing failure (low EV-H; shallow Ca^2+^ transients) [155]. Subsequently, following biology, are the corrections: When Ca^2+^ licensing is diminished, restore prostasome input or introduce engineered vesicles containing PMCA4/CD38 modules; when primary binding or IZUMO1 exposure is inadequate, incorporate head-targeting epididymosome-like fractions to reconstruct CRISP/ADAM/SPAM1 and PDIA/CLU support; when premature AR is prevalent, prioritise conservative anti-inflammatory management and NV clearance, and appropriately time capacitation signals [156].

### 5.5. Crosstalk with the Female Reproductive Tract

Subsequent to ejaculation, sperm navigate through a markedly different environment. Oviductal secretions, cervical mucus, and uterine fluid include many extracellular vesicles from the immune system and epithelium. These extracellular vesicles associate with sperm in a manner distinct to each domain, altering their behaviour in situ. Uterine and oviductal extracellular vesicles (uEVs/oviductal EVs) include tetraspanin-rich surfaces and phosphatidylserine/annexin interfaces that facilitate their attachment to the acrosomal cap, equatorial segment, neckpiece, and main piece [157]. These signals augment rather than facilitate the assimilation of previous epididymal and prostasomal inputs. Extracellular vesicles inside the female reproductive tract (oviductal/uterine) transport several components to sperm, including PMCA4 (a calcium efflux pump), SPAM1/PH-20 (hyaluronidase), oviductal glycoproteins that interact with glycans (such as OVGP1), lipid-modifying enzymes, and short RNAs [158].

Head-targeting lectins, PDIA-like oxidoreductases, and chaperones enhance receptor clustering and disulphide status on the apical membrane to facilitate zona recognition while preventing premature acrosome vesiculation [159]. The transfer of PMCA4, conversely, enhances Ca^2+^ homeostasis and refines the amplitude and decay of progesterone-induced transients, enabling the flagellum to transition seamlessly between hyperactivated and progressive modes. Simultaneously, EV membranes facilitate the transport of ceramide/lyso-lipid species and cholesterol-traffic machineries, which alter the order and curvature of rafts according to local pH/HCO_3_^−^, hence synchronising membrane fluidity with CatSper–PMCA4 microdomains. Vesicle-associated miRNAs (such as miR-34c-like and miR-21/30 clusters in experimental models) and tRFs engage with sperm ribonucleoprotein complexes, modulating kinase/ROS thresholds, which leads to a highly specific head–tail coordination dependent on location and timing as sperm transit from the uterine cavity to the isthmus and ampulla [47,158].

The immune epithelial mechanism facilitating this exchange safeguards allogeneic sperm and prepares the endometrium for implantation. Endometrial and cervical EVs include TGF-β, PTGS2/PGE_2_-associated mediators, and tolerogenic signals that alter the phenotypic of APCs and diminish the cytotoxicity of NK cells [160]. This alters the local immune system to a condition that is more amenable and regulated by inflammation. Their immunosuppressive components, including NK-interacting ligands and the expression of CD59/CD46, inhibit leukocyte activation and reduce oxidative stress in cervical mucus [161]. This maintains ROS association with capacitation within the “signalling, not damage” domain for an extended duration. Oviductal extracellular vesicles enhance a storage-release paradigm at the isthmic reservoir by maintaining head raft stability and acrosome integrity during dormancy, subsequently disrupting raft organisation and facilitating PMCA4 turnover in response to ovulatory signals [162]. This facilitates a rising progesterone gradient and cumulus signals to trigger controlled hyperactivation and, eventually, zona engagement at the ampulla. The functional total constitutes a bidirectional EV grammar: seminal vesicles influence immunological modulation and complement regulation to safeguard sperm enough for genetic modifications to occur, while uterine and oviductal vesicles dictate the time and intensity of capacitation, CatSper activation, and acrosome preparedness [163].

When one side of the discussion is biased, pathology is the inevitable result. Endometritis, cervicovaginal infection, or dysbiosis alters the uEV cargo, resulting in NF-κB/interferon signals. The complement-regulatory lipids are eliminated, and miRNAs that facilitate leukocyte recruitment and activate epithelial cells are introduced [164]. Upon entering this kind of mucus, sperm encounters elevated MPO/NOX activity, oxidised rafts, and truncated tetraspanin partners. This complicates vesicle docking and reduces the capacitation duration. Despite satisfactory numbers, early acrosome reaction, inadequate hyperactivation, and suboptimal fertilisation occur during IVF insemination [72]. Prostatitis and male reproductive tract inflammation induce a brief episode of hyperactivation that fails to penetrate the cumulus barrier and elicits inadequate progesterone responses. They also modify the surfaces of prostasomes to pro-inflammatory glycoforms and diminish the efficacy of Ca^2+^-licensing agents [165]. Conversely, excessively suppressive vesicle signals may diminish the essential antimicrobial response and increase the likelihood of infection dissemination. The presence of HPV, HSV, and HIV, together with subclinical viral excretion, alters the RNA and protein composition of seminal EVs and may initiate antiviral responses in the endometrium that are incompatible with conception, hence reducing receptivity and increasing the chance of miscarriage [166]. Sperm affected by endometriosis and chronic endometritis exhibit unstable acrosomes, abnormal phosphorylation kinetics, and impaired zona binding, whereas the endometrium does not achieve synchronised receptivity, even with viable embryos present. These settings result in the transformation of uEVs from tolerogenic/decidualising signals to pro-inflammatory prostaglandin/eicosanoid pathways. Prolonged storage at room temperature oxidises vesicle membranes and degrades targeting ligands, complicating their uptake by IUI/IVF media. Failure to eliminate CD45^+^ WBC-EVs introduces proteases that degrade PS–annexin interfaces critical for uEV docking. Semen processing that co-enriches NV lipoproteins randomly acquires cholesterol and induces premature capacitation prior to the stabilisation of the head by uterine EVs [167].

In ART, these blueprints transform into tangible activities. When integrated with uEV phenotyping from endometrial fluid or cervical mucus, fraction-aware profiling of spEVs (EV-H/M/L balance, prostasome markers, immune-modulatory signatures) can detect dyadic mismatches that impede empirical insemination, such as prostasome-deficient semen interacting with an inflamed endometrium. Integrating EV analytics with PLCζ status and head integrity assessments in ICSI helps in determining if AOA serves as an effective hedge or upstream conditioning. In IVF, adjusting the time of capacitation and progesterone exposure based on the patient’s EV signature prevents premature raft loss and acrosome reaction. In instances of prevalent male prostasomal deficits, reintroducing manufactured vesicles or EV-H containing PMCA4/CD38 and membrane-active scaffolds may reinstate Ca^2+^ licensing without inducing premature exocytosis. In cases marked by significant inflammation of the female reproductive tract, pre-treatment with antibiotics (where warranted) and anti-inflammatory protocols, together with leukocyte reduction, reduces exogenous oxidants and reinstates the composition of uEVs. Emerging media of oviductal or uterine extracellular vesicles, whether natural or biomimetic, may imitate physiological editing in vitro. This diminishes the likelihood of polyspermy and enhances motility persistence and acrosome stability. By analysing the bidirectional EV dialogue rather than exclusively depending on the semen analysis or endometrial thickness, clinicians can identify the primary lesion (immune tone, Ca^2+^ licensing, membrane priming, or receptor scaffolding) and choose interventions that synchronise sperm maturation with tract receptivity, thereby converting “idiopathic” fertilisation failure into a manageable, mechanism-based strategy.

## 6. Clinical Associations and Biomarkers: Linking Male Infertility Phenotypes and ART Outcomes to Sperm/Seminal Extracellular Vesicle Signatures

### 6.1. Asthenozoospermia: Clinical Correlations Derived from Semen Phenotype

In asthenozoospermia, the most reliable vesicle–phenotype signal is a change in the balance of seminal EVs from prostasome-rich EV-H (Ca^2+^ licensing) and/or antioxidant-dense EV-M to lipid-heavy EV-L and NV [168]. This manifests at the molecular level as a reduced delivery of Ca^2+^-targeted neckpiece modules (CD38/cADPR axis, PMCA4 scaffolding, and tetraspanin lattices) and a diminished GST/PRDX content per 10^6^ sperm equivalents. Even though total motility was “normal” at intake, the downstream physiology is easy to predict: shallow progesterone-evoked Ca^2+^ transients, flattened PKA/tyrosine-phosphorylation trajectories, curtailed hyperactivation, rising 4-HNE/MDA adducts on axonemal proteins, Δψm instability, and finally depressed VSL/VAP with erratic ALH/BCF under challenge. Fraction-aware proteomics usually lowers the CRISP/ADAM transfers that control head-tail crosstalk. In small-RNA panels, stress-pattern miRNAs are taking the place of let-7/miR-26/103/191 and redox-protective tRFs. In clinical practice, these signatures help tell the difference between damage-biased asthenozoospermia (EV-M low/high DFI/ROS), membrane-timing defects (EV-L excess with delayed cholesterol efflux), and signal-limited asthenozoospermia (EV-H low poor progesterone response) [116]. The probability of IVF fertilisation, blastulation, and recuperation from particular add-backs or antioxidants differs among each subtype. WHO classifications are not predictions of what will happen; they are based on references. This means that different labs and doctors may see the same category in different ways, which could change how they give advice and treat patients.

Teratozoospermia. Strictly morphologically, “minor” head and neck shapes often hide deficiencies in EV-encoded receptors and scaffolds [169]. Less head-targeting epididymosome cargo, like CRISP1, ADAM7, SPAM1, and PDIA/CLU chaperoning, leads to sparse or misfolded ZP-binding fields and an immature equatorial segment [170]. Changes in the ratios of tetraspanins (CD9/CD63/CD81) on vesicles or sperm also make it harder for microdomains to form. Timing errors in capacitation may be exacerbated by alterations in sperm/EV small RNAs, including the depletion of developmental tRNA-derived fragments (tRFs) and the accumulation of inflammation-associated miRNAs. These biases may also affect kinase and redox set-points in sperm (cAMP/PKA, PI3K/AKT, ROS management). Even though there is only a little bit of IZUMO1 exposure after AR, the hemizona binding is weak, and the AR timing is wrong, the counts and motility might be enough [171]. A head-ligand-low + EV-M-low profile suggests combined zona failure and embryo-stage risk from oxidative DNA damage. This means that capacitation control, antioxidant conditioning, or ICSI with a focus on AOA should be improved [172]. In practice, a head-ligand-low EV profile signifies suboptimal IVF fertilisation yet satisfactory ICSI results, particularly if PLCζ remains unaltered.

Oligozoospermia, or OAT. In bulk assays, low counts make it hard to tell the difference between signal and noise. EV measures not only quantify cells but also assess the secretory, inflammatory, and metabolic conditions of the tract, providing a sample-level (or “per-ejaculate”) evaluation of function. In essence, EV cargo and fraction balance contribute to traditional sperm parameters, including counts, motility, and morphology, by providing insights into the biology of the ejaculate. Mixed lesions that are often seen in OAT ejaculates include redox overload (EV-M depletion, lipid peroxidation products in spEVs), accessory-gland under-secretion (prostasome markers down), and the pattern indicates reduced epididymal input, as seen by lower levels of epididymosome-enriched proteins/miRNAs and a relative increase in testis-biased short RNAs in sperm or seminal extracellular vesicles (normalised per particles or protein) [173]. OAT characterised by maturation failure (low epididymal signal) can be differentiated from OAT characterised by post-ejaculatory failure (low prostasome signal) utilising composite panels that integrate fraction-aware protein features (such as GSTM2, PRDXs, CRISP1/ADAM7, and PMCA4) with miRNA/tRF ratios [174]. The first one predicts poor IVF insemination, even with rescued counts. If head ligands and small-RNA provisioning are very low, it may also not work well with ICSI. In vitro EV-H add-backs or changes to capacitation kinetics can usually fix the second one, which mainly affects progesterone responses and hyperactivation.

A “normal” semen analysis with a high DFI. EV readouts help to explain the common clinical problem of increased DNA fragmentation along with normal count, motility, and morphology. A Ca^2+^-void signature (prostasome-lean) leads to shallow progesterone responses and fertilisation failure with only modest DFI, whereas a redox-void signature characterised by EV-M depletion, low GSTM2 activity, peroxidised lipids in spEVs, and stress-biased miRNAs correlates with elevated TUNEL/SCSA and poor blastulation despite acceptable fertilisation. The first group benefits from EV-M-like supplementation and targeted antioxidant strategies (or choosing testicular sperm for ICSI when it cannot be fixed), while the second group benefits from timing capacitation to keep CatSper-PMCA dynamics or restoring prostasome input [175]. During the initial assessment, we must differentiate between the insights derived from quantitative metrics (sperm count, motility, morphology) and those obtained through an extracellular vesicle ‘liquid biopsy’ of the ejaculate. The first one counts cells, and the second one shows the molecular fingerprint of the ejaculate, which includes EV fractions, proteins, and short RNAs. For the classification of asthenozoospermia subtypes, the prediction of IVF fertilisation probability, and the identification of high-DFI risk, fraction-aware spEV panels consistently outperform raw semen parameters across phenotypes [176]. When pre-analytics and NV removal are meticulously regulated, protein characteristics (GSTM2/PRDXs for redox; CRISP1/ADAM7/SPAM1 for head function; PMCA4/CD38/tetraspanins for Ca^2+^ licensing) and small-RNA composites (let-7/miR-26/miR-200 families. tRF-Gly-GCC/tRF-Glu-CTC ratios) exhibit orthogonal signalling and endure cross-validation [177]. It is important that fraction identity is necessary, while precipitation techniques that co-enrich NV lead to false positives (premature capacitation signatures) and false negatives (loss of true EV-M antioxidant signal), collapsing EV-H/M/L masks pathognomonic shifts. Reporting a “EV signature” alongside WHO metrics, including EV-H low/PMCA4↓ (Ca^2+^-licensing deficit), EV-M↓/GSTM2↓ (redox lesion), and head-ligands↓ (zona axis), provides clinicians with a mechanism-anchored differential, reduces ineffective empiricism, and delineates specific subsequent actions (fraction-specific add-back, antioxidant conditioning, modified capacitation timing, ICSI ± AOA, or TESE consideration when upstream production is the predominant signal).

### 6.2. Azoospermia & Spermatogenesis (NOA vs. OA; TESE Prediction)

Azoospermia is not only an issue with sperm count; it represents a dysfunction throughout the whole reproductive system. To ascertain whether the failure originates from manufacturing, maturation, or distribution, EVs convert that binary output into a map of upstream biology [178]. In OA, the spEV pool is biased towards prostasome-rich EV-H with preserved Ca^2+^-licensing signs (PMCA4/CD38/tetraspanin lattices) due to ongoing accessory gland secretion and obstructed flow from the epididymis. However, because to the physical obstruction of the caput/cauda’s luminal flow, the spEV pool exhibits diminished signals from the epididymosome/testis [126]. The proteome contains several robust glandular scaffolds; yet there is a scarcity of head-targeting ligands (CRISP1/ADAM7/SPAM1) and oxidoreductases/chaperones often included during transit. This pattern indicates proficient ejaculation but little post-testicular modification [144]. In NOA, testicular output is compromised. Semen often displays a “gland-only” EV profile marked by increased stress and immunological indicators (Annexins, S100s, acute-phase markers) and less cargo derived from the germline, SSC, and epididymis [179]. Small RNA panels in spEVs demonstrate the distinction: Prostasomal miRNAs persist, leading to an elevated gland-to-epididymal ratio that is undetectable with standard semen analysis, whereas the epididymal accumulation of miRNAs/tsRNAs decreases in NOA (let-7/miR-26/miR-191/miR-200 families; tRF-Gly/-Glu signatures) [52]. Orthogonal proteome indicators validate origin: despite robust prostate output, the lack or significant decrease in germ cell-associated traits (such as TEX101-lineage signals and testis-biased peptides) and epididymal chaperones/oxidoreductases (CLU, PDIA, and PRDX/GPX modules) indicates upstream production or maturation deficiencies [180]. This categorises “azoospermia” into two separate routes for counselling and intervention: OA-like (obstructive azoospermia with retained spermatogenesis) and NOA-like (non-obstructive azoospermia characterised by production or maturation failure with inflammatory changes).

Extracellular vesicles serve as a non-invasive indicator of active spermatogenesis, aiding in the identification of lesions along the testis epididymis gland axis for testicular sperm extraction planning in non-obstructive azoospermia [181]. The probability of retrieval increases when spEVs retain testis/epididymis signatures, including developmental miRNA/tRF motifs, chaperones/oxidoreductases, germline-associated peptides (TEX101-pathway surrogates), and ligands targeting the epididymal head (CRISP/ADAM/SPAM1). This is especially relevant in instances of hypospermatogenesis and late maturation arrest, when localised sperm production continues. A “prostasome-only + inflammatory” profile, characterised by significant EV-H, reduced epididymal/testis signals, heightened leukocyte-EV contamination, or NV burden, indicates a low retrieval yield and necessitates pre-harvest optimisation. This includes endocrine correction (FSH/LH/thyroid/androgen axes), anti-inflammatory management, mitigation of febrile/oxidative stress, and varicocele repair when necessary, prior to surgical intervention. The prediction performance improves when EV analytics are integrated with endocrine and imaging data [182]. For example, fraction-aware spEV panels including FSH, inhibin B, and testis ultrasound/volume increase AUCs enhance TESE prediction and prevent unnecessary microTESE procedures in patterns only seen in Sertoli cells. Refrain from using precipitation methods that co-enrich NV to avert false positives resembling epididymal cargo. Furthermore, retain the fraction identity, since compressing EV-H/M/L masks diminishes low-abundance germline signals among prostasome noise [182]. The straightforward trio of (i) existence of testicular/epididymal extracellular vesicles, (ii) strength of glandular extracellular vesicles, and (iii) inflammation/neovascularisation load provides a rapid and valuable assessment of non-obstructive azoospermia vs. obstructive azoospermia and the likelihood of sperm retrieval by testicular sperm extraction [178]. This facilitates the formulation of surgical choices based on molecular data rather than just on hormonal factors.

These changes are linked to the functionality of the tract and are rational from a mechanical perspective. Spermatogenesis transpires in OA, leading to the production of epididymal vesicles that are proximally ensnared. As a result, semen has a “prostate-heavy” composition, characterised by preserved Ca^2+^ licensing and reduced maturation alterations in the ejaculate. This corresponds with positive ICSI results when surgically obtained sperm are available [183]. In NOA, Sertoli-Leydig communication is disrupted; germ cell EV transport is reduced. Sertoli cell EV miRNAs that govern steroidogenesis and preserve the niche are dysregulated, and the ejaculate exhibits immunological activation and interstitial stress rather than maturational modification [184]. Pathology-specific impressions augment the image: Hypoxia related to varicocele induces HIF-associated stress miRNAs and oxidised lipids in spEVs. Gonadotoxins and chemotherapy diminish the transport of small RNA and chaperones in the epididymis. Endocrine insufficiency alters the production of prostasomes and glyco-codes, complicating the regulation of Ca^2+^ even in the presence of a limited number of spermatozoa [52]. These indicators predict trajectory while also identifying aetiology: Established “gland-only + immune” patterns indicate poor retrieval odds and direct couples towards donor options or alternative timeframes, whereas profiles marked by reversible stress (varicocele, inflammation) predict EV normalisation and an enhanced TESE yield after correction. Electric vehicles assist physicians in correlating pathology, prognosis, and procedure by transforming azoospermia from an enigmatic phenomenon into a molecular map with fractions. This enables them to evade unnecessary surgery and missed opportunities for recovery.

### 6.3. ART Outcomes (Fertilisation, Embryo Development, Pregnancy, Live Birth)

Stage of fertilisation. EV signals forecast the point of cycle disruption during insemination by indicating the status of the head-scaffold and Ca^2+^-licensing mechanisms. Despite sufficient numbers and morphology, hyperactivation remains incomplete due to prostasome-deficient EV-H profiles, inadequate neckpiece-targeting ligands, attenuated sAC → cAMP → PKA phosphorylation kinetics, and diminished progesterone-induced Ca^2+^ transients [185]. Insufficient head ligands (CRISP1/ADAM7/SPAM1) from epididymal EVs result in a sluggish zona approach, unequal hemizona binding, poor PMCA4 recycling, insufficient ABHD2 alleviation of the 2-AG inhibition, and early stabilisation of tyrosine phosphorylation levels in these samples [186]. Rescue is time-sensitive and mechanistic: raft dynamics are maintained for a long enough duration to enable effective docking when EV-H or modified vesicles containing PMCA4/CD38 and lipid-active scaffolds are reintroduced during or soon before to capacitation. This restores the amplitude and decrease of Ca^2+^ without precipitating action potential generation prematurely. Upstream vesicle conditioning aims to restore head receptor clustering and acrosome constraint to baseline levels; however, if extracellular vesicle data and PLCζ status indicate an oocyte-activation impediment (PLCZ1-deficient, mislocalised, or variant), fertilisation may still be salvaged by transitioning to ICSI ± AOA [187].

In summary, fraction-aware EV readouts (EV-H/M/L balance + head-cargo intensity) assist in determining whether to continue with IVF using prostasomal rescue, transition to ICSI, and/or implement layer AOA. This transforms the ambiguous danger of “failed fertilisation” into a definitive strategy for the subsequent phase of the embryo. Cycles that achieve fertilisation but experience stalling between days three and five are often attributed to EV-M depletion coupled with redox overload, rather than entrance failure. These samples often exhibit reduced small-RNA profiles for epididymal tRF/miRNA sets that promote early cleavage, heightened DFI, and oxidised lipids in spEVs [52]. Biology is consistent: diminished GSTM2/PRDX levels let reactive oxygen species degrade rafts, convert polyunsaturated fatty acids into 4-HNE/MDA, and crosslink protamines. Consequently, fertilisation may occur (PLCζ and Ca^2+^ licensing are enough); however, cleavage symmetry and blastulation are disrupted by the kinetics of the first cell cycle and the load of DNA repair [188]. Correctives must be customised to the lesion: employ media that stabilise Δψm and maintain raft integrity; modulate capacitation rates to reduce oxidative spikes (enhanced regulation of albumin/HCO_3_^−^; prevent NV-rich precipitation that initiates premature cholesterol efflux); and sustain or augment EV-M (native or engineered GST/PRDX/Trx modules). A diminished epididymal RNA library indicates the potential for premature arrest, despite normal semen characteristics. This information aids in obtaining permission, determining the number of embryos to use, and providing the option to transition to testicular sperm if the systemic oxidative burden cannot be rectified [114]. Small RNA composites derived from spEVs that consider fractions are associated with the quality of blastocysts and the symmetry of timelapse measures. In conclusion, the restoration of antioxidant vesicles and a more refined capacitation mechanism, rather than just relying on generic antioxidants, are essential for addressing day-3/5 failures associated with EV-M-low signatures in clinical pregnancy and implantation [189].

Implantation links the extracellular vesicle ecologies of men and females. Decreased complement regulators (CD46/CD59), modified NK-interacting ligands, and a lack of prostasomes represent immunomodulatory defects in seminal EVs that impair post-coital immunological tolerance and reduce the safe capacitation period in cervical mucus in males [190]. During the exportation of miRNAs that facilitate leukocyte recruitment and epithelial signalling, uEV patterns on the female side, influenced by endometritis/dysbiosis, endometriosis, or chronic inflammation, often enhance NF-κB/interferon pathways, hence eliminating tolerogenic signals and complement control. Inconsistent ERA-positive cycles are expected to correlate with inferior clinical pregnancy outcomes, reduced implantation rates, or biochemical losses [191]. A combined profile (semen extracellular vesicles + urinary extracellular vesicles) addresses male prostasomal immunology (restoring EV-H; treating prostatitis/dysbiosis; timing intercourse/IUI/ET during quiescent periods) and female endometrial inflammation (administering antibiotics when necessary, implementing anti-inflammatory protocols, correcting the microbiome) or both. Fraction-aware spEV panels may be used in conjunction with endometrial markers to develop a dyadic, mechanism-based implantation approach. They consistently outperform WHO criteria in predicting fertilisation, blastulation, and pregnancy due to their incorporation of Ca^2+^ licensing, redox regulation, membrane priming, and receptor scaffolding [192]. A concise EV signature, such as EV-H↓ (Ca^2+^ axis), EV-M↓ (redox), head-ligands↓ (zona axis), and CD45+ contamination↑ (inflammation), indicates the specific side or sides requiring treatment prior to transfer.

The post-thaw function is an EV-dependent phenotype due to freeze-thaw inducing phase shifts, collapse of leaflet asymmetry, and bursts of reactive oxygen species that stress the structures stabilised by extracellular vesicles [193]. Incorporating EV-M-like preparations or designed vesicles containing GSTM2/PRDX/Trx before to chilling (to preload membranes and elevate GSH/GSSG) and/or immediately post-thawing (to mitigate the burst) decelerates the increase in DFI, maintains Δψm stability, and preserves hyperactivation capability [194]. Upon freezing, samples exhibiting EV-M deficiencies accumulate lipid peroxidation and chromatin damage. Restoring the CatSper–PMCA4 microdomains affected by cold shock and introducing prostasomes post-thaw reinstates progesterone responsiveness; controlled exposure prevents premature AR. The methodology is essential. Select DGC and controlled-rate freezing, eschew precipitation that co-enriches NV (which depletes cholesterol and induces premature capacitation), and contemplate a rapid EV-H/M co-incubation post-thawing to rebuild neck/head microdomains before to the progesterone challenge [195]. These modifications are GMP-compliant, compatible with standard DGC/swim-up methodologies, and transform a general “post-thaw quality” issue into a precise vesicle reintroduction and management protocol that safeguards biological integrity membrane organisation, Ca^2+^ licensing, redox balance that governs fertilisation, blastulation, and live birth outcomes [196].

### 6.4. Infection and Inflammation Contexts (HPV/HSV/HIV, Prostatitis, Leukocytospermia)

The first post-ejaculatory time and the length of isthmic residency in the female reproductive canal are referred to as the “shortened capacitation window.” In oxidative and inflammatory circumstances, such as leukocytospermia, chronic prostatitis/CPPS, and oxidative stress associated with varicocele, capacitation-related signalling often starts prematurely and diminishes rapidly. This increases the likelihood of premature acrosome activation prior to reaching the ampulla. By “infection or sterile inflammation reprogram EV cargo,” we refer to active inflammatory conditions occurring during semen generation and, where applicable, in peri-ovulatory tract secretions. The ejaculate’s and the tract’s extracellular vesicles exhibit these conditions by possessing a reduced quantity of stabilising and antioxidant components, with an increased presence of inflammatory proteins and miRNAs. In leukocytospermia, activated neutrophils and monocytes release ROS and HOCl bursts, as well as NET debris, and flood semen with CD45^+^ WBC-EVs that contain proteases (such as elastase and MMPs), myeloperoxidase, and cytokines [197]. This environment cuts the tetraspanin partners, PS–annexin bridges, and MFGE8–integrin contacts that EVs use to dock, carbonylates zona-interaction proteins, and oxidizes PUFA-rich head rafts and principal-piece lipids. This leads to less productive docking, bad vesicle fusion, and early AR, while protamine crosslinking and aldehyde adducts make DFI go up at the same time. Prostatitis/dysbiosis diminishes pH-regulated fusion at the neckpiece, downregulates complement regulators (e.g., CD46/CD59) and calcium-licensing mechanisms (PMCA4/CD38 scaffolds), and alters prostasome glyco- and proteo-signatures to pro-inflammatory variants. In clinical terms, this shows up as weak progesterone responses, temporary hyperactivation, and poor IVF insemination performance even though the counts are fine. Viral exposures add different EV fingerprints [198]. High-risk genotypes correlate with diminished motility and elevated DFI, aligning with EV-mediated oxidative stress and head-scaffold degradation. This change makes embryos less receptive, shortens the immune-quiet window after insemination, and makes the timing for capacitation stricter, even with good embryos. Exposures to HSV/HIV change the immune cargo of seminal EVs to interferon/NF-κB programs. This sends out miRNAs and proteins that make the cervico-uterine environment more antiviral, but it also makes the decidual environment less ready [199]. We adhere to consensus recommendations for suspected inflammation or infection of the male genitalia. This includes managing fever or systemic inflammation, conducting a focused microbiological investigation, and administering targeted antibiotics or anti-inflammatories as necessary. Semen collection and assisted reproductive technology are deferred until semen parameters stabilise and clinical resolution is achieved. Prostate exosomal protein in urine serves as an EV-based biomarker associated with the severity of prostatitis; however, its correlation with ART timing remains unverified [200]. Human Papillomavirus (HPV) in semen might diminish sperm motility and fertilisation potential. Routine sperm washing may not entirely eliminate HPV, potentially diminishing sperm-zona interactions and adversely affecting ART results. HPV binds to the sperm head via syndecan-1. It is unclear whether HPV particularly modifies the seminal-EV miRNA/proteome; nonetheless, alterations in seminal-EV cargo linked with illness have been recorded in several situations [201]. Mistargeted or underpowered EV–sperm interactions (head docking, neck licensing) coupled with an excessive oxidant load constitute the prevalent molecular pathology in these scenarios. All of these things together make it harder to find the right time to do productive capacitation, AR, and zona engagement.

When viewed through an EV lens, these signals can be read and acted upon. High CD45^+^ EV burden indicators, along with a “pro-inflammatory prostasome” signature that shows low complement-regulatory display, changed tetraspanin ratios, and less PMCA4/CD38 content [202]. In the laboratory, stringent regulation of HCO_3_^−^/albumin/pH, NV control (utilising SEC/iodixanol in lieu of precipitation), and leukocyte reduction (meticulous DGC → swim-up) safeguard EV-mediated Ca^2+^ licensing, inhibit premature cholesterol efflux, and maintain head rafts. uEV profiling from cervical/uterine fluid aids in timing embryo transfer during an immune-quiet period and directs the treatment of endometritis/dysbiosis in cases of predominant female-tract inflammation. Prostasome-lean semen confronting inflamed uEVs advocates for ICSI rather than IVF insemination to prevent a compromised zona axis [203]. For HPV-positive semen with persistent abnormalities, strict selection (DGC + swim-up to reduce virus-bearing debris/EVs), fraction-aware antioxidant support (restore EV-M function; quench ROS to protect head rafts), and ICSI should be used. When zona-binding is consistently poor, priority should be given to controlling inflammation and maintaining fraction integrity during HSV/HIV exposure, allowing residual prostasomes to license CatSper without inducing premature acrosome reaction [204]. This short EV report replaces vague “oxidative stress” labels with specific plans for each mechanism: treat inflammation first, restore missing fractions (EV-H for Ca^2+^, EV-M for redox), avoid precipitation artefacts, and retime capacitation [205]. Table 5 presents a succinct decision matrix linking EV signatures to stage-specific dangers and corresponding actions.

## 7. Therapeutic and Interventional Horizons: Restoring Missing EV Functions

The fundamental concept is to temporarily restore the absent vesicle modules in the sample as required, rather than to create new biological entities. When the semen EV signature is devoid of Ca^2+^ characterised by low PMCA4/CD38 levels in EV-H with shallow, slowly fading progesterone-induced Ca^2+^ transients briefly incubating sperm with their own EV-H prior to capacitation constitutes the first stage [206]. Titrate in your laboratory until a consistent increase in the amplitude of the Ca^2+^ peak and a more rapid decline are seen in live imaging. Subsequently, an increase in hyperactivation within the same medium should be seen. Utilise the sperm count rather than volume to determine the dosage [207]. The exposure duration should be measured in minutes, not hours. This duration is sufficient to repair the CatSper–PMCA4 microdomains at the neckpiece but not too prolonged to cause premature depletion of cholesterol in the head. Gentle and rapid washing will preserve the edit and eliminate loose vesicles prior to albumin/HCO_3_^−^ inducing capacitation [208]. If autologous EV-H is unavailable or does not pass sterility testing, a screened donor EV-H produced under GMP-like circumstances may be used as an alternative. However, this is only feasible after ensuring that the add-back does not induce off-target acrosome reactions and that the potency aligns with your own Ca^2+^ test.

When redox governance is the primary issue, characterised by low GSTM2/PRDX levels in EV-M, unstable Δψm, and elevated ROS and DFI the remedial method involves EV-M supplementation either immediately before capacitation or immediately after thawing for cryopreservation cycles [209]. A subdued oxidative baseline is preferable to a pronounced Ca^2+^ increase in this instance. This indicates reduced DCF/MitoSOX signals, an elevated GSH/GSSG ratio, and a more stable Δψm with progesterone exposure [210]. Clinical success is achieved at the embryo stage, characterised by improved cleavage symmetry and enhanced day-5 yield, even when fertilisation is already satisfactory. To prevent redox rescue from being exchanged for premature cholesterol efflux, integrate EV-M supplementation with membrane-preserving media (restricted albumin; regulated HCO_3_^−^), since it functions as a mobile antioxidant barrier [211]. Maintaining natural EV-L connections throughout preparation and moderating the capacitation ramp (albumin/HCO_3_^−^ dosage, pH elevation) is more critical than instinctively introducing EV-L to samples exhibiting membrane-timing discrepancies (excessive head rafts, postponed cholesterol efflux, misaligned AR). Exercise caution when reintroducing EV-L directly, since excessive raft reinforcement may further impede efflux and disrupt the acrosome timing during zona interaction.

It is essential to implement each add-back in accordance with the method rather than personal belief. Prior to clinical use, EV-H lots must demonstrate verified PMCA4 activity and CD38 levels, together with appropriate Ca^2+^ amplitude and decay in a validated sperm imaging assessment [126]. In a bench test predicting reduced ROS/DFI in the partner’s sperm, EV-M lots must demonstrate GST activity and prevention of lipid peroxidation. Head-centric fractions resembling epididymosomes should exhibit enhanced visibility. Exposure to IZUMO1 at the equatorial segment, a more stringent AR window on FITC-PSA/PNA, and the reestablishment of hemizona binding. Potency is limited by duration and frequency of usage; upon seeing physiological rescue, cease exposure, rinse, and continue. Each validation must include negative controls, such as heat-inactivated vesicles and NV-enriched fractions, to demonstrate that the observed impact is unique to vesicles rather than an artefact of lipoprotein or protein aggregates [212].

Safety is an area of academic inquiry. Eliminate gradient residues from any lot that may interact with gametes (iodixanol interferes with epitopes and CatSper functionality), verify sterility, ensure endotoxin and mycoplasma negativity, and minimise exposure durations to prevent premature capacitation or spontaneous acrosome reaction [213]. Avoid “treating through” inflammation with add-back in cases of leukocytospermia or active infection; rather, diminish the inflammatory burden by clinical treatment and leukocyte reduction, and re-profile EVs. Oxidants and proteases oxidise lipid rafts and cleave docking ligands, hence reducing the capacitation window regardless of the supplements administered. Viral proteins and RNAs may coalesce and alter the immunological response inside the endometrium [214]. Consequently, use EV for diagnostics only in individuals with HPV, HSV, or HIV when adhering to IRB-sanctioned, pathogen-mitigated methods. Select your ICSI cycles judiciously: Head-ligand add-back is often unnecessary; however, EV-M pulses during thawing or preparation might mitigate the increase in DFI post-thaw. A micro-dose of EV-H just before to ICSI may be rational if Ca^2+^ regulation is minimal and AOA is not required, but only after your laboratory has evaluated safety and efficacy [215].

Anticipate and systematically direct those who fail to react. If the EV-H add-back fails to enhance Ca^2+^ amplitude or reduce decay despite rigorous pre-analytics, consider intrinsic variations in CatSper, ABHD2, or PMCA4, and opt for ICSI ± AOA rather than increasing dosage or duration [216]. Assess testicular sperm for ICSI while mitigating systemic oxidative damage and addressing upstream variables such as varicocele, fever, smoking, and metabolic syndrome, particularly if EV-M support stabilises Δψm but DFI remains high [217]. If hemizona binding and AR timing remain suboptimal after head-focused add-back, proceed with ICSI for this cycle and defer head-axis rehabilitation for subsequent IVF efforts. To preserve the intervention’s mechanism of action and clinical validity, timestamp each step related to capacitation, standardise dosage based on sperm concentration (reporting particles per 10^6^ cells), and establish predefined objectives (Ca^2+^ rescue for EV-H, ROS/Δψm/DFI for EV-M, hemizona/AR for head-fractions) [72].

## 8. Discussion

This narrative review presents a fraction-aware framework for sperm-derived EVs, which explicitly links the origin and cargo of vesicles to distinct functional axes, including membrane priming/timing, redox regulation, Ca^2+^ licensing and clearance, and zona-interaction scaffolding. These axes are later associated with clinically detectable phenotypes throughout the fertilisation, embryonic development, and implantation phases. Three recent human investigations have implemented and enhanced this methodology. To extract NV material, Wang, Zhu, Tang, Zhou, and Li originally fractionated seminal EVs into EV-H, EV-M, and EV-L using high-resolution iodixanol gradients [102,137,206,218,219]. Each fraction has distinct proteomes and functions that do not intersect. For instance, EV-H enhances motility and capacitation, EV-M provides antioxidant capacity via GSTM2, while NV independently induces an early acrosome response. Luo, Zhu, Liu, and Tan discovered a method to isolate CD63+/CD52+ epididymosomes directly from ejaculates by recognising CD52 as an epididymis-specific marker and used flow cytometry for sorting [218,220,221]. This is a viable human approach for source specificity. The sorted population markedly improved sperm function relative to other CD63^+^ seminal EV subsets, indicating that source-pure vesicles provide better biological results [222]. Third, using rigorously regulated functional co-incubations, Tamessar, determined that bulk SFEVs somewhat improve human sperm motility in particular contexts and do not independently trigger acrosomal exocytosis or capacitation [223]. Our analysis reveals that primitive small-EV pellets derived from semen include vesicles from many anatomical origins, including the prostate, epididymis, and sperm. Therefore, a significant fraction of the sfEV signal in these samples presumably originates from several non-sperm-specific sources.

Compelling evidence currently exists for the mechanistic consistency of epididymal contributions across various techniques. Barrachina et al., used human-mouse integrative analysis to find 25 conserved sperm proteins derived from the epididymis [173]. Subsequently, high-resolution confocal imaging was used to demonstrate that four proteins SLC27A2, EDDM3B, KRT19, and WFDC8 were localised in the epididymal epithelia rather than in the seminiferous tubules [173]. Fluorescently tagged epididymosomes physically engaged with sperm and enabled the transfer of cargo to designated domains. Mouse systems proteomics further refines the understanding: Skerget, Rosenow, Petritis, and Karr documented segmental proteomes, indicating that about one-third of proteins associated with known sperm abnormalities are incorporated during epididymal transit [224]. This enhances cauda sperm’s motility and sperm-egg identification capabilities while documenting dynamic changes in immunity-related proteins during development. Luo and colleagues’ development in human sorting employs similar reasoning by recognising CD52 as a suitable epididymosome marker in ejaculates and demonstrating the augmented functional importance of the CD63+/CD52+ population [220]. This resolution is essential for clinical translation to develop decision-grade assays capable of distinguishing between epididymal and accessory-gland signals in real time, as well as for head-axis rehabilitation.

The density-resolved EV-H/EV-M/EV-L architecture on the prostate side, as documented by Wang and associates, provides a quantitative foundation for prostasomes and corresponds with their recognised neckpiece-oriented roles [137]. Antioxidant enzymes, such as GSTM2 and peroxiredoxins, are prevalent in EV-M, which mitigates intrinsic ROS and preserves Δψm under capacitation stress. EV-L is lipid-rich and probably contributes to raft organisation and membrane timing regulation at the head-neck interface [225]. EV-H comprises Ca^2+^-handling proteins, including PMCA4 and CD38, which modulate the magnitude and duration of progesterone-induced CatSper transients, allowing hyperactivation without an immediate acrosome response [226]. Two urgent clinical ramifications exist. In the absence of PLCζ, a Ca^2^-void signature (reduced EV-H Ca^2+^ levels) signifies fertilisation-stage failure, despite adequate sperm numbers and motility, hence supporting prostasome-aware intervention or a shift to ICSI ± AOA [42]. A redox-void signature (poor EV-M antioxidant capability coupled with elevated lipid peroxidation) forecasts day-3/5 arrest, despite sufficient insemination. This indicates the need for antioxidant vesicle support and a more delicate approach to capacitation. Lin and Liang conclusively revealed by iTRAQ proteomics that the seminal EV proteome is altered in asthenozoospermia, notably showing a substantial decrease in TRPV6 levels in ASEVs and ejaculated sperm [37,214]. This reveals a definitive molecular link between EV cargo and motility phenotype, validating the idea that vesicle makeup predicts semen-level functioning.

Small RNAs provide an additional, independent measure that is both diagnostically reliable and physiologically consistent. Research conducted by Vojtech et al., indicates that human seminal exosomes include a distinctive array of short non-coding RNAs [227]. This includes Y RNAs, tRNA-derived fragments (tRFs) enriched for 5′ ends at certain lengths that might block translation, and a multitude of miRNAs, characterised by a limited, conserved dominant subset among donors. This donor-stable environment is just what a clinical test requires [228]. Zhou, Xiao, and Chen demonstrated that seminal exosomal miR-210-3p levels increase with varicocele severity, exhibit a negative correlation with sperm count and inhibin-B, and decrease after microsurgical varicocelectomy [76,102,221]. This likely indicates Sertoli cell damage associated with hypoxia, which may be monitored non-invasively as part of timing assessments. Pathology impressions might be analysed on the same layer. The observations, coupled with the testis-level insights obtained by Ma, and Chen concerning the dynamic roles of testis-derived EVs in spermatogenesis and steroidogenesis specifically signals from macrophages and Sertoli cells that can penetrate the blood–testis barrier and be identified in semen formulate a mechanistic framework for identifying intratesticular stress in spEV small-RNA panels, which has immediate implications for the timing of ART [35,76].

Our research establishes the parameters, while the technique serves as the critical determinant of the validity of the findings. Wang and colleagues demonstrate that iodixanol gradients may efficiently segregate EV-H, EV-M, and EV-L from NV. If NV is not eliminated, functions are incorrectly allocated [229]. Only the NV portion induced the acrosome response, a significant artefact that might erroneously associate premature AR with “EVs” if gradients or SEC are not used. Luo and co-authors use a supplementary technique called flow sorting, using CD63 triggering and CD52 selection, to extract a population of human epididymosomes from the ejaculate [222]. This eliminates a significant source of misunderstanding in mixed pellets and enhances the biological signal. Conversely, polymer-based precipitation procedures may co-enrich NV substances, such as lipoproteins and protein–RNA complexes, and facilitate particle/protein aggregation, which may complicate vesicle-specific analyses. According to Ma et al., these stages are used in some segments of the varicocele workflow [38]. This approach is effective for discovery and establishing linkages; nevertheless, it must be used cautiously when clinical assertions or functional attributions rely on pristine vesicles. Tamessar and colleagues emphasise the significance of the functional assay’s design [230]. Bulk SFEVs promote modest increases in motility of high-quality sperm in pH-controlled, realistic conditions, and do not independently trigger capacitation or acrosomal exocytosis [231]. These results agree precisely with (i) a diverse origin in crude pellets, (ii) the loss of fraction identity, and (iii) a significant percentage of seminal EV signalling directed towards the female tract, which aligns with the immunomodulatory roles attributed to prostasomes.

This fraction-aware map has immediate therapeutic implications. The integration of fraction-resolved proteomics/sncRNA panels with concurrent physiological assessments, such as progesterone-induced Ca^2+^ imaging and ROS/Δψm stability, enables stage-specific risk evaluations. These encompass: EV-H low → fertilisation risk, EV-M low → embryo risk; and head-axis depletion in epididymosome cargo (including chaperones and zona ligands) → zona-binding risk [23]. The most viable short-term therapeutic solution involves the native, temporally restricted reintroduction of the absent module: a brief exposure to EV-H to restore Ca^2+^ transients in Ca^2+^-deficient signatures; a short EV-M pulse during capacitation or thawing to diminish ROS/DFI in redox-deficient signatures; and, if accessible, exposure to CD63^+^/CD52^+^ epididymosomes for head-axis anomalies, which Luo and colleagues noted surpass generic CD63^+^ EVs in efficacy [222]. When integrated with proteomic indicators, such as the absence of epididymal chaperones, seminal extracellular vesicle small-RNA composites from the Vojtech-style landscape, in conjunction with epididymal/testicular signals as delineated by Ma and colleagues, can facilitate micro-TESE triage in azoospermia and differentiate between OA and NOA [227]. To reduce superfluous IVF tries and synchronise intervention with biological processes, the miR-210-3p varicocele research by Ma et al. introduces a preliminary corrective checkpoint monitoring the normalisation of the EV signal before cycles that depend on accurate capacitation timing [38].

The synthesis’s strengths lie in the convergence of orthogonal lines: density-resolved human EV biology, source-pure epididymosome isolation, mechanistic transfer to sperm and functional assays conducted under realistic conditions [173,222,223,229]. It unambiguously differentiates EVs from NV, resolving previous disputes over “EV-induced” acrosome alterations. Nonetheless, it is crucial to recognise the constraints. Numerous human datasets are cross-sectional and limited in size; dyadic datasets that associate semen EVs with uterine/oviductal EVs within the same cycle are rare, constraining causal relationships between male EV signatures and implantation biology [55]. Furthermore, human epididymal validation often depends on cross-species inference due to the challenges in acquiring epididymal fluid. Furthermore, although Parrella et al., demonstrated that PLCζ-centred mechanisms predominantly regulate oocyte activation failure, there is a deficiency of human studies examining whether the reestablishment of fraction-specific seminal EV modules can affect late gamete-interaction processes in a PLCZ1-sensitive manner [232]. Ultimately, genetic constraints exist: vesicle rescue may cease to function if the CatSper subunits, ABHD2, ADCY10, PMCA4, or PLCZ1 undergo independent alterations. The diagnostic algorithm must include EV signatures with genotype and swiftly convert to ICSI ± AOA or to testicular sperm as necessary.

Future efforts should concentrate on four distinct avenues addressing each of the aforementioned translational obstacles. To ensure that EV-H/EV-M/EV-L calls align and synchronise single-particle fluorescence with MESF scales across several instruments, multi-site ring trials with shared, fraction-resolved reference materials are used. A “low EV-H” call in one laboratory will have the same implication in another laboratory. (i) The uniform implementation of CD52-based sorting, as outlined by Luo et al., to provide human epididymosome preparations for standardised functional tests, including zona-synchronised IZUMO1 exposure, hemizona binding, and acrosome response time, to quantitatively rectify the impaired head axis. (ii) Prospective, stage-specific trials that randomly allocate native, short-exposure add-back to patients pre-selected based on the pertinent EV signature and assess on-target rescue: hemizona/AR timing for epididymosomes, ROS/Δψm/DFI for EV-M, and Ca^2+^ amplitude/decay for EV-H; to maintain attribution, negative controls should comprise heat-inactivated vesicles and NV-enriched fractions. (iii) Paired spEV–uEV profiling during cycles to elucidate the biology of fertilisation and implantation an area where the extensive immunomodulatory literature on prostasomes and the Tamessar findings (subtle SFEV effects on sperm) indicate a vital yet inadequately comprehended male–female interface. 

## 9. Conclusions

Semen analysis is the first assessment, despite the fact that sperm functioning is determined by membranes, ion channels, redox thresholds, and receptor scaffolds, which are not detectable by standard counts and motility evaluations. Extracellular vesicles derived from sperm and seminal fluid provide a distinct and dynamic link between genetic disorders and clinical phenotypes. EV-H assesses Ca^2+^ licensing at the neckpiece, EV-M evaluates antioxidant capacity and Δψm resilience, EV-L analyses membrane order and timing, while head-focused epididymosome cargo indicates the condition of zona-binding architecture when fractional identity is maintained and contamination is regulated. Recurrent implantation failure, day-3/5 arrest, and “idiopathic” fertilisation failure may be addressed as manageable issues when associated with same-day functional complements, allowing for stage-specific treatments.

The algorithm is user-friendly. Obtain a fraction-aware EV signature during consumption, accompanied by WHO measures. Subsequently, under standardised capacitation settings, conduct two rapid functional assessments: ROS/Δψm stability and progesterone-induced Ca^2+^ imaging. Proceed with IVF insemination and a brief EV-H rescue if the profile is devoid of Ca^2+^ but maintains redox integrity. If Ca^2+^ levels remain constant, transition to ICSI and consider AOA when PLCζ is diminished. When the systemic oxidative load becomes permanent, evaluate testicular sperm and expect improvements at the embryonic stage. If the profile is redox-void, stabilise with EV-M-like support during capacitation or thaw and limit cholesterol efflux. Prioritise ICSI for the present cycle while restoring the head axis upstream in case of head ligand depletion and unpredictable AR timing. If the membrane timing is problematic, retain the EV-L connections and adjust the albumin/bicarbonate concentrations to align with the acrosome’s preparation for interaction with the zona. In the presence of contamination or inflammation, as seen by increased CD45^+^ and ApoA1 levels, prioritise therapy, followed by isolation, and defer insemination rather than continue based on a misdiagnosis. Following significant physiological alterations upstream, such as a complete spermatogenic cycle for testicular restoration or many weeks for inflammatory and endocrine management, use extracellular vesicle trends rather than inadequate measurements of quantity or morphology to validate the subsequent endeavour.

The primary emphasis in the subject now lies in rigorous standards and outcome-oriented validation. Fraction-aware EV diagnostics and precise, time-sensitive interventions will improve fertility when Ca^2+^ licensing is the limiting factor, increase day-5 yield when redox is constrained, maintain implantation when immune tone is misaligned, and prevent unproductive cycles when upstream biology is insufficiently prepared. They will not replace ART; rather, they will enhance its safety and personalisation. The practical commitment is a reduction in inexplicable failures, expedited access to appropriate therapies, and a more explicit correlation between molecular illness and successful live births.

## Figures and Tables

**Table 1:** Sperm-relevant extracellular vesicles by anatomical source: markers, cargo, sperm targeting, functions, and clinical associations.

Origin/Tissue	EV Subpopulation (Example)	Surface Markers (Indicative)	Key Cargo (Indicative)	Sperm Domain Targeted	Core Functions (Biological Axis)	Typical Clinical/Pathological Associations
Testis (SC, germ cells, Leydig)	Testis-derived EVs	CD9/CD63/CD81; annexin V; (less source-specific markers)	miR-486-5p, miR-145-5p; PDIA/CLU; NO/steroidogenesis signals	SSC niche/seminiferous tubules; may traverse BTB	SSC support; regulation of maturation/steroidogenesis; niche homeostasis	Varicocele-linked signatures (e.g., miR-210-3p); NOA reduction in testicular signal; toxicant/pyrexia imprints
Epididymis (caput/corpus/cauda)	Epididymosomes	MFGE8/ITGB1, CD9/CD63/CD81	CRISP1, ADAM7, SPAM1, PDIA, CLU, AKAPs; glycolytic/redox enzymes; tRF/miRNA	Head (acrosomal cap/equatorial segment), neck, principal piece	Maturation; assembly of zona-receptor/fusogen complexes; chaperoning/disulphide editing; metabolic support	Deficit → weak hemizona binding, premature/erratic AR, poor IVF fertilisation despite normal counts
Prostate	Prostasomes (EV-H/EV-M/EV-L by density)	PSCA/GLIPR2, CD46/CD59, CD9/CD63/CD81	PMCA4; CD38 (cADPR); Ca^2+^/lipid-active enzymes; complement regulators; PRDX/GST (notably in EV-M)	Neckpiece/midpiece (CatSper–PMCA); head (stabilisation)	Ca^2+^ licensing to progesterone; immunoregulation/anti-complement; oxidative protection	EV-H deficit → shallow Ca^2+^ peaks/sub-hyperactivation. EV-M deficit → high DFI/Δψm instability. Prostatitis/dysbiosis → pro-inflammatory profile.
Seminal vesicle	SV-EVs/protein-rich secretions	CD9/CD63 (general); FN1/SEMG-related content	Semenogelins/proteases; lipids modulating viscosity/flow; antimicrobial peptides	Semen milieu/mucosal interface	Viscosity/liquefaction; chromatin/membrane protection; regulation of cervical-mucus interaction	Excess viscosity or protease imbalance → trapping, delayed capacitation, secondary oxidative injury

Synthesis of the principal EV populations by origin (prostate, testis, epididymis, and seminal vesicle), including representative cargo (proteins, lipids, and sncRNAs), characteristic surface markers, the sperm domains they target, critical functions, and prevalent clinical/pathological associations that facilitate interpretation and decision-making. The results are marked with words like “Human,” “Mouse,” “Cat,” “Boar,” and so on. Non-human data are presented for translational and mechanistic context.

**Table 2:** The fraction-resolved proteomic modules of semen extracellular vesicles and their clinical and functional correlations.

Source/Fraction	Representative Cargos (Examples)	Sperm Domain Targeted	Primary Function	Loss-of-Function Phenotype	Lab Readout (Same-Day)	Mechanism-Matched Action
Epididymosome (caput → cauda)	CRISP1/4, ADAM7, SPAM1, PDIA, CLU, MFGE8, TCP1/CCT, AKAP3/4, GAPDHS, LDHC	Head (acrosomal cap/equatorial), neck, principal piece	Receptor assembly, zona interaction, disulphide editing, chaperoning; ATP supply	Weak hemizona binding, unstable AR timing, suboptimal hyperactivation with normal counts	Hemizona/IZUMO1 exposure; capacitation tyrosine-P; CASA hyperactivation	Preserve/add epididymosome-like fraction; prioritise ICSI when head-axis remains deficient
Prostasome EV-H	PMCA4, CD38/cADPR axis, lipid-active enzymes, tetraspanins	Neckpiece–CatSper–PMCA microdomains	Ca^2+^ licensing and rapid signalling to progesterone	Shallow Ca^2+^ transients; delayed/weak hyperactivation; IVF fertilisation drop	Live Ca^2+^ imaging (progesterone pulse); PMCA activity	Short, timed EV-H add-back; IVF → ICSI ± AOA if PLCζ also low
Prostasome EV-M	GSTM2, PRDXs, TRX modules	Midpiece/principal piece	Redox buffering; Δψm stability; chromatin protection	High ROS/DFI; day-3/5 arrest despite fertilisation	DCF/Δψm assays; SCSA/DFI	EV-M add-back peri-capacitation or post-thaw; adjust capacitation to reduce ROS spikes
Prostasome EV-L	Lipid-dense scaffolds (raft formers), annexins	Head–neck membranes	Membrane priming and timing	Over-ordered heads; delayed cholesterol efflux; mistimed AR	Cholesterol efflux kinetics; AR timing with P4/ionophore	Moderate albumin/HCO_3_^−^; avoid excessive EV-L carryover; combine with EV-M support
NV (non-vesicular)	Lipoproteins, protein/DNA complexes	N/A	Can mimic capacitation cues; artefact risk	Premature AR; spurious capacitation readouts	AR assays (spontaneous rise); lack of vesicle markers	Remove via SEC/DG; do not ascribe function to NV

**Table 3:** sncRNA panels in seminal EVs: directionality, phenotypes, and clinical utility.

EV Source/Fraction	Dominant sncRNAs (Examples)	Direction in Pathology	Associated Phenotype	Assay & Specimen	Clinical Use
Epididymosome-enriched spEV	let-7, miR-26/103/191/200 families; tRF-Gly-GCC/tRF-Glu-CTC	↓ in tract inflammation/metabolic stress	Early embryo arrest; impaired capacitation timing	qPCR/NGS on fractioned spEV (EV-ID kept)	Prognosis for blastulation; select adjuvant EV-M; counsel timing
Prostasome-lean EV-H	miRNAs modulating Ca^2+^ cascades	↓ in Ca^2+^-void signatures	Fertilisation failure at IVF	qPCR on EV-H band	Decide EV-H add-back vs. IVF → ICSI ± AOA
Redox-focused EV-M	miRNAs targeting ROS/Δψm regulators	↓ in high DFI	Day-3/5 failure	qPCR on EV-M band	Peri-capacitation EV-M supplement
Testis-biased signals	miR-210-3p and hypoxia-responsive sets	↑ in varicocele; ↓ post-repair	Sertoli stress; low count/inhibin-B	qPCR from whole semen spEV	Monitor repair; triage TESE timing
Azoospermia classifiers	mixed miRNA/tRF composites	Distinct NOA vs. OA patterns	Residual spermatogenesis	qPCR/NGS on spEV	Non-invasive TESE prediction

The dominant miRNA/tRF families in semen extracellular vesicles and epididymosomes, their evolutionary trends in common diseases, associated symptoms, test techniques, and clinical applications (diagnosis/prognosis). Terms such as “Human,” “Mouse,” “Cat,” “Boar,” and others denote the outcomes. Non-human data are included for translational and mechanistic perspective.

**Table 4:** Characteristics of lipid and metabolite profiles in semen extracellular vesicles: biophysical features, pathogenic markers, and remediation strategies.

Fraction	Dominant Lipids/Metabolites	Biophysical Role	Pathology Signature When Altered	Practical Readouts	Corrective Strategy
Epididymosome	SM, cholesterol, PC/PE/PS/PI (segment-specific acyl chains), gangliosides; polyamines, carnitine, lactate/pyruvate	Stabilise head rafts; prepare for controlled efflux; support ATP microdomains	Over-ordered heads; delayed capacitation; waveform instability	Laurdan/merocyanine; cholesterol efflux kinetics; CASA	Tune albumin/HCO_3_^−^; preserve epididymosome contacts; avoid harsh DGC
EV-H	Cholesterol-transfer proteins; lipases; CD38/cADPR tools	Rapid post-ejaculatory licensing; P4-responsive Ca^2+^ transients	Shallow Ca^2+^ rise/slow decay; IVF fertilisation drop	Live Ca^2+^ imaging; tyrosine-P kinetics	Timed EV-H add-back; consider ICSI ± AOA if PLCζ low
EV-M	GSTM2, PRDX5/6; TRX axis; PUFA-protection	Suppress ROS; stabilise Δψm and chromatin	High ROS/DFI; day-3/5 arrest	DCF/Δψm; SCSA; 4-HNE/MDA adducts	EV-M pulse; redox-aware capacitation; antioxidant media
EV-L	Lipid-dense tubular scaffolds	Head–neck priming and docking stability	Delayed cholesterol efflux; mistimed AR	AR timing assays; raft order	Limit EV-L excess; balance with EV-M

The role of key lipid classes and metabolic buffers in membrane organisation and curvature, the expected inadequacies when reduced or excessive, relevant laboratory assessments, and suggested corrective measures are all discussed in the context of EV fractions. Terms such as “Human,” “Mouse,” “Cat,” “Boar,” and others signify the results. Non-human data are included for translational and mechanistic insights.

**Table 5:** EV Signatures ⇒ Stage-Specific Risk → Recommended Actions in ART.

Stage at Risk	EV Signature (Fraction-Aware)	Expected Problem	Recommended Countermeasure	Expected Benefit	Notes/Pitfalls
Fertilisation (IVF)	Low EV-H; weak Ca^2+^ tools	Shallow P4 Ca^2+^ transients; poor hyperactivation	Short EV-H add-back; or pivot to ICSI ± AOA if PLCζ low	↑ 2PN rate	Keep exposure minutes; verify sterility/endotoxin
Cleavage/Blastulation	Low EV-M; peroxidised lipids	High ROS/DFI; D3–5 arrest	EV-M supplementation; redox-aware capacitation/media	↑ Day-5 yield; better symmetry	Monitor Δψm; avoid nonspecific antioxidants alone
Zona interaction/AR timing	Head-axis depletion (epididymosome cargo ↓) or EV-L excess	Weak hemizona; mistimed AR	Preserve epididymosome contacts; moderate albumin/HCO_3_^−^	Improved insemination performance	Consider ICSI if persistent
Implantation	Pro-inflammatory prostasome pattern; uEV inflammation	Low receptivity	Treat male inflammation; manage female uEV/endometritis	↑ Implantation CPR	Use dyadic profiling when feasible
Cryosurvival	EV-M deficit	Post-thaw ROS spike/DFI ↑	EV-M pulse pre-/post-thaw	Better post-thaw hyperactivation	GMP feasibility; batch QC

A decision-grade matrix that connects fraction patterns to the stage of failure that is most likely to happen (fertilisation, cleavage/blastulation, cryosurvival, or implantation), as well as the expected benefits and recommended steps to avoid failure.

## Data Availability

No new data were created or analysed in this study. Data sharing is not applicable to this article.

## Abbreviations

AOA	assisted oocyte activation
CASA	computer-assisted sperm analysis
CatSper	cation channel of sperm
DFI	DNA fragmentation index
DG/DGUC	iodixanol) density-gradient ultracentrifugation
DLS	dynamic light scattering
dUC	differential ultracentrifugation
EV(s)	extracellular vesicle(s)
IFC	imaging flow cytometry
IVF/ICSI	n vitro fertilisation/intracytoplasmic sperm injection
MISEV	Minimal Information for Studies of Extracellular Vesicles
NOA/OA	non-obstructive/obstructive azoospermia
NTA	nanoparticle tracking analysis
NV	non-vesicular nanoparticles (particles lacking a delimiting membrane; e.g., lipoproteins and protein/protein–RNA complexes; often co-isolate unless removed by density gradients or SEC)
OAT	oligo-astheno-teratozoospermia
OEV/OVS	oviductal extracellular vesicles/oviductosomes
PMCA4	plasma-membrane Ca^2+^-ATPase 4
PLCZ1	phospholipase C zeta 1
ROS	reactive oxygen species
SEC	size-exclusion chromatography
spEV(s)	seminal extracellular vesicle(s)
TESE	testicular sperm extraction
TEM/cryo-EM	transmission electron microscopy/cryo-electron microscopy
Δψm	mitochondrial membrane potential
